# Supplementary material for: Delivering genome sequencing in clinical practice: an interview study with healthcare professionals involved in the 100 000 Genomes Project
Source: BMJ Open. 2019 Nov 3;9(11):e029699. doi: 10.1136/bmjopen-2019-029699 (PMC6858183; doi:10.1136/bmjopen-2019-029699)
Supplement: Supplementary data [file bmjopen-2019-029699supp001.pdf]

Study invitation sent to potential participants

Dear [name] ,

As you are aware we are running a research study looking at patient’s views and experience of being offered whole genome sequencing through the 100,000 Genomes Project. As part of this study it is important to understand health professional experiences and views around offering whole genome sequencing. This research will help us to develop educational material for health professionals and for patients considering whole genome sequencing.

We would like to invite you to take part in an interview. If you would be willing to help with the study we will ask you to be interviewed either face-to-face at your place of work or by phone, whichever suits you. The interviews take around 30-45 minutes and will cover points such as your experience of discussing sequencing, consenting patients and delivering results and the potential barriers to informed consent and how can these be minimised.

The discussion will be audio-taped, the transcripts will be anonymised and your name will not be used in any reports or publications. Participation is, of course, voluntary and you can change your mind about being in the study at any time, without giving a reason. This research is organised by Research Teams at Great Ormond Street Hospital and UCL Institute of Child Health. The study is funded by the National Institute for Health Research.

If you are willing to take part in an interview or want to ask further questions, please let us know either by email or telephone.

If you would like any more information please let us know.

We look forward to hearing from you.

Interview guide for healthcare professionals

Background of participant and 100KGP training

1. What is your professional background? e.g. cardiologist, paediatrician etc
2. Had you offered whole genome sequencing in either a research or clinical capacity prior to the 100,000 Genomes Project beginning?
3. Can you briefly describe your role in the 100,000 Genomes Project?
4. Prior to recruiting participants into the 100,000 Genomes Project, did you have to undergo any type of training? If so what training did you undergo?

Recruitment into the 100KGP

5. Which of your patients do you discuss the 100KGP with?
6. Can you describe the discussion you have with those patients?
   a. What do you tell them about the 100KGP?
   b. What questions do they ask about the 100KGP?
   c. How long does that discussion tend to last?
   d. Have there been any difficulties you have encountered whilst discussing the 100KGP with patients?
7. If the patient is interested in taking part in the 100KGP what do you do next?
8. Have you had many people that decline to be referred into the 100KGP?
   a. What are their reasons for declining?
9. Have you come up against any problems recruiting into the study e.g. organisational issues or other difficulties?

Return of results in the 100KGP

10. Have you returned any results from the 100KGP to your patients?

    If participant HAS returned results

    a. Can you describe how you receive those results?
    b. What do you do once you have received those results? (write to patient? ask them to come to a clinic appointment?)
11. What kinds of results have you given to patients? (Primary findings; secondary findings)
12. What has the experience of returning results from WGS been like for you?
    a. Do you feel adequately prepared to discussion WGS results with patients?
    b. Have you encountered any difficulties?

c. Are you comfortable returning secondary findings?

13. What are patients' reactions to receiving results?

    a. What type of questions to patients ask?

    b. What concerns if any do they have?

    c. Do you feel able to address any concerns that they have?

If participant HAS NOT returned results

14. Do you know how the results will be sent to you?

    a. How will you contact the patient?

    b. Do you feel adequately prepared to discuss WGS results?

    c. Are you comfortable with returning secondary findings?

    d. Can you anticipate any difficulties?

Moving WGS into routine clinical practice

15. What do you think about whole genome sequencing being offered in routine clinical practice after the 100,000 Genomes Project has been completed?

    a. What do you see as the main advantages, benefits?

    b. What concerns, if any, do you have about offering whole genome sequencing being offered in routine clinical practice?

    c. In your view, what (if any) are the main limitations of using whole genome sequencing for routine clinical practice?

    d. In your view, what (if any) are the main challenges of offering whole genome sequencing in routine clinical practice?

16. Is there anything you would like to change about how whole genome sequencing is offered?

17. What advice would you give to other healthcare professionals discussing whole genome sequencing with patients?

18. What are your thoughts about how we would train health professionals that might be expected to offer whole genome sequencing in the future?

Interview guide for consenters

Background of participant and 100KGP training

19. What is your professional background? e.g. genetic counsellor, research fellow etc
20. What experience did you have of offering genetic testing or sequencing to patients prior to your involvement in the 100,000 Genomes Project?
    a. Specifically, had you offered whole genome sequencing prior to the 100,000 Genomes Project beginning?
21. Do you have any other relevant experience that you have brought to the role, e.g. consenting patients for research?
22. Can you briefly describe your role in the 100,000 Genomes Project?
23. Prior to recruiting participants into the 100,000 Genomes Project, what training did you undergo?
    a. Training specifically provided by the 100,000 Genomes Project
    b. Other training
24. What are your thoughts on the training that you did?
    a. Do you have any thoughts about the way it was delivered?
    b. Did you feel it adequately prepared you to recruit into the 100KGP?
    c. Did you feel anything was missing / could be improved?

Recruitment into the 100KGP

25. What has been your experience of recruiting patients into the 100KGP?
    a. What aspects of recruitment have worked well?
    b. What aspects of recruitment haven't worked well?
    c. How might recruitment be improved in your opinion?
26. Have you come up against any other problems recruiting into the study e.g. organisational issues or other difficulties?

Assessing informed consent in the 100KGP

27. What is your approach to consenting patients into the 100KGP?
    a. How do you generally begin the conversation?
    b. Are there set ways you conduct the session i.e. when you go through the consent form, family history etc or does it alter depending on the participants?

c. Does the discussion tend to be led by you or by the patient?

28. When you are talking to patients about consenting to the 100,000 Genomes Project, do you try to explore their understanding of the Project?

    a. If yes, how do you do this? Do you use any particular techniques to elicit patients' knowledge and understanding? What are they?

29. When you are talking to patients about consenting to the 100,000 Genomes Project, do you try to explore their attitudes, views, preferences, concerns around the Project?

    a. If yes, how do you do this? Do you use any particular techniques to elicit patients' attitudes and/or concerns? What are they?

30. What (if any) do you think are the main barriers to helping patients make informed choices about taking part in the 100,000 Genomes Project? (length of appointment; cultural barriers; lack of tools to support the consent discussion)

31. How does the method of contact i.e. whether they know about the 100KGP when they arrive at the hospital or whether they are recruited unexpectedly following another appointment, impact the consent discussion?

    a. Does it make a difference if they have already received written information about the study prior to the consent appointment?

Participants experiences in the 100KGP

32. What are the common questions patients have about taking part in the study?

    a. What concerns do patients have about taking part?

    b. What are their reasons for declining to take part?

33. Which are the main aspects of the study that you feel you spend most time discussing with patients (e.g. what genome sequencing is, secondary findings, accessing data etc)?

34. Do you think patients are putting a lot of thought into the decision to accept/decline testing? Do any want additional time to think about it?

35. What has been your experience discussing secondary findings with patients?

    a. Is it something that generates discussion and if so what kind of discussion?

    b. Who is the discussion between? (patient / parents themselves? Patient and health professional?)

36. Have you had experience of receiving consent or assent from a patient under the age of 16? What has been your experience?

37. Have you had to reconsent anyone turning 16 to remain in the project (with capacity)? What has been your experience of doing this?

    a. Has anyone withdrawn?

Moving WGS into routine clinical practice

38. What do you think about whole genome sequencing being offered in routine clinical practice after the 100,000 Genomes Project has been completed?

    a. What do you see as the main advantages, benefits?

    b. What concerns, if any, do you have about offering whole genome sequencing being offered in routine clinical practice?

    c. In your view, what (if any) are the main limitations of using whole genome sequencing for routine clinical practice?

    d. In your view, what (if any) are the main challenges of offering whole genome sequencing in routine clinical practice?

39. Is there anything you would like to change about how whole genome sequencing is offered?

40. What advice would you give to other healthcare professionals discussing whole genome sequencing with patients?

41. What are your thoughts about how we would train health professionals that might be expected to offer whole genome sequencing in the future?
