# Supplementary material for: Delivering genome sequencing in clinical practice: an interview study with healthcare professionals involved in the 100 000 Genomes Project
Source: BMJ Open. 2019 Nov 3;9(11):e029699. doi: 10.1136/bmjopen-2019-029699 (PMC6858183; doi:10.1136/bmjopen-2019-029699)
Supplement: Author's manuscript [file bmjopen-2019-029699.draft_revisions.pdf]

BMJ Open is committed to open peer review. As part of this commitment we make the peer review history of every article we publish publicly available.

When an article is published we post the peer reviewers' comments and the authors' responses online. We also post the versions of the paper that were used during peer review. These are the versions that the peer review comments apply to.

The versions of the paper that follow are the versions that were submitted during the peer review process. They are not the versions of record or the final published versions. They should not be cited or distributed as the published version of this manuscript.

BMJ Open is an open access journal and the full, final, typeset and author-corrected version of record of the manuscript is available on our site with no access controls, subscription charges or pay-per-view fees (http://bmjopen.bmj.com).

If you have any questions on BMJ Open's open peer review process please email info.bmjopen@bmj.com

BMJ Open

Delivering genome sequencing in clinical practice: an interview study with healthcare professionals involved in the 100,000 Genomes Project

Journal:	*BMJ Open*
Manuscript ID	bmjopen-2019-029699
Article Type:	Research
Date Submitted by the Author:	21-Feb-2019
Complete List of Authors:	Sanderson, Saskia; Great Ormond Street Hospital For Children NHS Foundation Trust, NE Thames Regional Genetics Service; University College London<br>Hill, Melissa; Great Ormond Street Hospital For Children NHS Foundation Trust, ; NE Thames Regional Genetics<br>Patch, Christine; King's College London<br>Searle, Beverly; Unique - Understanding Rare Chromosome and Genetic Disorders<br>Chitty, Lyn; NE Thames Regional Genetics; UCL, Institute of Child Health<br>Lewis, Celine; NE Thames Regional Genetics; UCL
Keywords:	QUALITATIVE RESEARCH, genome sequencing, next-generation sequencing, MEDICAL EDUCATION & TRAINING, interviews

SCHOLARONE™
Manuscripts

Delivering genome sequencing in clinical practice: an interview study with healthcare professionals involved in the 100,000 Genomes Project

Saskia Sanderson[1,2], Melissa Hill[1],* Chris Patch[3,4,5], Beverly Searle[6], Celine Lewis[1],** Lyn Chitty[1]

*Joint first and corresponding author

** Joint last author

[1] North East Thames Regional Genetics Service, Great Ormond Street Hospital for Children NHS Foundation Trust, London, UK

[2] Institute of Health Informatics, University College London, London, UK

[3] Florence Nightingale Faculty of Nursing and Midwifery, King's College London, London, UK

[4] Genomics England, Queen Mary University of London, Dawson Hall, London, UK

[5] Counselling, Society and Ethics Research, Wellcome Genome Campus, Cambridge, UK

Key words: *genome sequencing, next-generation sequencing, medical education and training, qualitative research, interviews*

Corresponding authors

Dr Saskia Sanderson and Dr Melissa Hill

NE Thames Regional Genetics Service

Great Ormond Street Hospital

Level 5, Barclay House

37 Queen Square

London WC1N 3BH

Email: saskia.sanderson@ucl.ac.uk or Melissa.hill@ucl.ac.uk

For peer review only

ABSTRACT

Objectives: Genome sequencing is poised to be incorporated into clinical care for diagnoses of rare diseases and some cancers in many parts of the world. Healthcare professionals are key stakeholders in the clinical delivery of genome sequencing-based services. Our aim was to explore views of healthcare professionals with experience of delivering genome sequencing via the 100,000 Genomes Project.

Design: Interview study using thematic analysis.

Setting: Four NHS hospitals in London.

Participants: Twenty-three healthcare professionals (5 genetics clinicians and 8 non-genetics clinicians (all Consultants), and 10 'consenters' from a range of backgrounds) involved in identifying or consenting patients for the 100,000 Genomes Project.

Results: Most participants expressed positive attitudes towards genome sequencing in terms of improved ability to diagnose rare diseases, but many also expressed concerns, with some believing its superiority over exome sequencing had not yet been demonstrated, or worrying that non-genetics clinicians are inadequately prepared to discuss genome sequencing results with patients. Several emphasised additional evidence about utility of genome sequencing in terms of both main and secondary findings is needed. Most felt non-genetics clinicians could support patients during consent, as long as they have appropriate training and support from genetics teams. Many stated genetics experts will play a vital role in training and supporting non-genetics clinicians in variant interpretation and results delivery, particularly for more complex cases.

Conclusions: Healthcare professionals responsible for delivering clinical genome sequencing have largely positive views of the sequencing in terms of improving diagnostic yield, but also significant concerns about practical aspects of delivery. Non-genetics clinicians delivering genome sequencing require guidance and support. Additional empirical evidence is needed to inform policy and practice, including: how genome compares to exome sequencing; utility of secondary findings; training, in particular of non-genetics health professionals; and mechanisms whereby genetics teams can offer appropriate support to their non-genetics colleagues.

For peer review only

STRENGTHS AND LIMITATIONS OF THIS STUDY

- The qualitative methodology employed in this study meant that the sample size was small, but allowed in-depth exploration of interviewees' perspectives and allowed them to express their views in their own words
- Some of the interviewees were 'consenters' from the 100,000 Genomes Project so although they had real experience of supporting patients during the informed consent process, most will not be delivering genome sequencing clinically in the future
- Interviewees also included a range of genetics and non-genetics healthcare professionals from multiple hospital sites who *will* be responsible for delivering genome sequencing in the new UK Genomic Medicine Service
- Interviewees' views were informed by their experience of actually offering genome sequencing to NHS patients, rather than views being based only on hypothetical scenarios only
- Our findings shed important light on where the evidence gaps about clinical genome sequencing are from healthcare professionals' perspectives; this information may also be of use to practitioners and policymakers concerned with delivering genome sequencing clinically going forwards

For peer review only

BACKGROUND

Genome sequencing (the process of determining most of a person's complete set of DNA) is being incorporated into clinical care for the purposes of diagnosing rare disease and some cancers. In the National Health Service (NHS) in England, the 100,000 Genomes Project recruited patients with rare diseases and their relatives, and patients with cancer, for genome sequencing between 2015 and 2018. In England, as part of the new NHS Genomic Medicine Service, genome sequencing will shortly be offered as part of this new service. In England as elsewhere, there are efforts to mainstream genomic testing such that, when indicated, both genetic and non-genetic clinicians will be able to order genome sequencing for their patients, as they would other clinical tests. The main stated benefits of genome sequencing for rare disease diagnoses include increased discovery rate of causative gene variants, the potential to dramatically shorten the 'diagnostic odyssey', preventing multiple, often invasive and expensive, investigations and allowing targeted therapies.[1] However, moving genome sequencing from research to clinical practice raises many challenges. These include how to ensure there is the relevant expertise and infrastructure to deliver clinical genome sequencing nationally; whether non-genetic clinicians need support to offer genome sequencing and return results to patients as genomics is mainstreamed, and if so, what kind of support; and what results should be returned and to whom?

Healthcare professionals are key stakeholders in answering these questions given they are central to offering genome sequencing to patients, facilitating informed consent, supporting patients' decision-making, and returning results to patients. Previous studies have explored healthcare professionals' experiences of obtaining informed consent for genomic sequencing [2-4] and returning genomic results,[5 6] and views regarding secondary findings from genomic sequencing.[7 8] Most of these studies were conducted in the US [2-7], one in Canada[8] and the only UK one to date drew on experiences of a single Multi-Disciplinary Team (MDT) and focused exclusively on secondary findings, defined as DNA variants identified through sequencing that have implications for a person's health or that of their offspring but are not related to the original clinical indication for sequencing.[9]

For peer review only

Here we aim to explore views of a range of health care professionals involved in the 100,000 Genomes Project regarding the potential clinical delivery of genome sequencing for rare disease diagnoses in the new Genomic Medicine Service, with a view to providing insights that may be relevant to policy and practice.

METHODS

Study design

A qualitative approach using one-to-one interviews for data collection was used to facilitate in-depth exploration of healthcare professionals' viewpoints on moving genome sequencing into NHS clinical practice.

Ethical approval

Ethical approval for this study was obtained from the West Midlands NHS Research Ethics Committee (15/WM/0258).

Patient and public involvement

The advisory team for this study had one lay-person and three patient support group representatives; including co-author BS, who has contributed to the revision of the manuscript.

Sampling and recruitment

Participants were recruited from four London hospitals that were part of two Genomic Medicine Centres (GMCs) recruiting affected individuals and their relatives into the 100,000 Genomes Project. Three groups of health professionals were recruited to the study: (1) genetic and (2) non-genetic clinicians involved in identifying patients eligible to take part in the 100,000 Genomes Project; and (3) 'consenters', i.e. members of the 100,000 Genomes Project consenting teams whose role included consenting patients for genome sequencing. Potential participants in this study were identified by our research team and invited to participate via an email which included participant information explaining the aims of the study. They were asked to contact the researchers (CL, MH, SS) if interested in participating.

Participants were purposefully sampled across several different London hospitals to ensure inclusion of all locations, various approaches to recruitment, and clinicians from a range of clinical backgrounds. Consenters

For peer review only

in the 100,000 Genomes Project have a range of backgrounds, including genetic counselling, research, and other post graduate training. All consenters are trained, including taking the online course, “Preparing for the consent conversation” (https://www.genomicseducation.hee.nhs.uk/courses/courses/consent-ethics/).

Interviews

An interview guide was developed for this study by the investigators to explore participants’ experiences and views on (a) recruitment into and (b) return of results in the 100,000 Genomes Project, as well as their views on moving genome sequencing into clinical practice, and their professional background and training for the 100,000 Genomes Project. To develop the interview guide, a first draft of a set of questions reflecting the aims of the project was drafted by one of the investigators (MH) with experience in qualitative research methods; this was iteratively refined via discussion with two other investigators (CL, SS) on the team also with experience and expertise in qualitative methods; minor revisions were then made to the interview guide in response to questions that emerged during the first two interviews. There were two versions of the interview guide: one for clinicians and one for consenters. The consenter version included additional sections about views on more specific aspects of informed consent and patients’ experiences (see Supplemental Material). The interviews lasted 30-45 minutes and were conducted face-to-face or via telephone by CL, MH or SS.

Data Analysis

Interviews were digitally recorded, transcribed verbatim and anonymised. Pseudonyms were then assigned to each participant. Data were analysed using the principles of thematic analysis [10]. NVivo version 10 (QSR International, Pty Ltd, Australia) software was used to facilitate coding and data analysis.

The research team comprised three postdoctoral researchers (S.C.S., C.L., M.H.) with expertise in conducting qualitative analyses, two genetics health-care providers (C.P., L.S.C.) with experience supervising qualitative analyses, and one patient advocate (B.S.). The transcripts were coded by S.C.S. and M.H. The two investigators independently coded the same transcript and developed a draft codebook, and the two versions of the codebooks were compared and combined into a single codebook. The analyses focused primarily on text segments relevant to moving genome sequencing for rare disease diagnosis into clinical practice, reflecting the aims of this study. The two investigators then independently coded a second transcript: a Kappa was calculated and indicated good inter-rater reliability. From this point the two investigators independently coded the remaining transcripts into eight broad overarching categories. The overarching category specifically

For peer review only

focused on moving genome sequencing into routine clinical practice was then coded line by line into meaningful units of text. To ensure rigor and increase authenticity, multiple investigators (S.C.S., C.L., M.H.) with varying levels of familiarity with genome sequencing checked the emerging themes at multiple timepoints and together grouped the themes to form broader themes that were reviewed and redefined as the analysis progressed.

RESULTS

Participants

The 23 participants were 5 genetics clinicians (all Consultants in clinical genetics), 8 non-genetics clinicians (all Consultants, from a range of specialities including audiology, paediatric nephrology, neurology, and paediatrics), and 10 consenters (with a range of backgrounds, including research nursing, project management, postdoctoral research, and medical doctor trainees) from four hospital sites. See Table 1 for additional participant characteristics.

Overarching themes

Participants' views on moving genome sequencing for rare disease diagnosis into clinical practice fell into three broad overarching themes as described below.

1. Attitudes towards moving genome sequencing into clinical practice

1.1. Perceived benefits

Participants talked about three main ways in which clinical genome sequencing will benefit patients. First, some participants felt that clinical genome sequencing will lead to improved diagnoses for patients with rare diseases by making the path to diagnosis more efficient, making it more likely patients will get a diagnosis with fewer tests needed. Second, some participants felt that clinical genome sequencing will add to the knowledge base about rare diseases. Third, several participants talked about how research will lead to improved treatments for future patients, either by improving disease progression stratification or by personalising treatments based on medication responsiveness (pharmacogenomics). See Table 2 for illustrative quotes.

For peer review only

1.2. Concerns

Although many participants expressed positive attitudes towards genome sequencing, some also raised concerns. First, a few participants expressed concern that genome sequencing has not been demonstrated to be superior to exome sequencing for diagnosing rare diseases, and believed genome sequencing should not be introduced clinically until there is scientific evidence about its value. Second, many participants had concerns about informed consent, particularly around the time needed for this. Participants emphasised that the consent form for the 100,000 Genomes Project was very long, and moreover that consent discussions had been undertaken by a dedicated consent team who would no longer be available after the transition to clinical practice. Some were concerned that some non-genetics clinicians do not understand the limitations of genome sequencing sufficiently, and that they are therefore under-prepared to offer genome sequencing to their patients, e.g.

> *"…having overheard conversations from colleagues, I don't think they fully understand the limitations on what they are offering. I think clarity as to what this test will tell you, what it will not tell you. I think that needs to be clarified. And I think the colleagues need to understand before they offer it to their patients."* (Participant 12, genetics clinician)

Third, participants voiced concerns about adequate resources – including laboratory and clinician time and expertise – being available to analyse and interpret sequence data and subsequent variant validation, with several feeling genetics departments will be "swamped". Participants also raised concerns about phenotyping taking up the time of non-genetics clinicians, e.g.

> *"presumably we would still have to do the phenotype data and certainly for clinicians, that's the limiting step, filling out another form!"* (Participant 23, non-genetics clinician)

Concerns were also expressed about the potentially very long turn-around times to results. Several participants were also concerned that reports generated by labs were not "filtered" enough, meaning a lot of work was still left for clinicians to do. Fourth, a few participants expressed concerns about disclosing results to patients both in terms of the amount of consultation time needed to discuss results with patients and families, and whether non-genetic clinicians have insufficient understanding to be able to interpret and return more complex results. See Table 2 for additional illustrative quotes.

For peer review only - http://bmjopen.bmj.com/site/about/guidelines.xhtml

2. Attitudes towards offering secondary findings from genome sequencing

2.1. Perceived benefits

Participants perceived three main benefits of offering secondary findings from genome sequencing in clinical practice. First, several participants felt that offering clinically actionable secondary findings in the clinical setting will provide the opportunity to identify patients at increased disease risk, allowing for improved disease prediction, prevention or early diagnosis. Second, several participants felt that the clinical delivery of secondary findings will lead to research learning opportunities: for example, how we will learn more about the impact of secondary findings on patients. Third, several participants felt that patients want or would want to receive secondary findings, suggesting that offering this type of information might be responsive to patients' desires and preferences. See Table 3 for illustrative quotes.

2.2. Concerns

The issue of whether secondary findings should be offered to patients divided participants, with some advocating a cautious adoption whilst collecting data on penetrance and outcomes, and others stating that we should not be offering secondary findings at all. First, some were concerned that evidence is lacking regarding the penetrance of variants identified "completely incidentally" and that we may be "over-estimating penetrance" as a consequence.

Related to this, the second sub-theme that emerged was a concern that evidence is lacking regarding whether it is "clinically useful" to return secondary findings to patients, particularly if they do not have a known family history of the associated condition. One participant said that this "opens a can of worms".

The third sub-theme that emerged was concern about the potential psychological impact of the results on participants, such as causing "anxiety". One participant asked, "How do you stop people from suffering unduly from getting these results back?" (Participant 8, consenter), while another asked, "are you opening up Pandora's box?" (Participant 5, consenter). One participant likened secondary findings to picking up aneurisms incidentally on brain scans (see Table 3).

The fourth sub-theme that emerged was a specific concern that non-genetic clinicians are not prepared to discuss secondary findings. One participant was concerned that doctors without "a genetics training background" might misinterpret secondary findings and that this could lead to "false reassurance" (Participant 6, consenter). Another emphasised that non-genetic clinicians couldn't decide whether mutations

For peer review only - http://bmjopen.bmj.com/site/about/guidelines.xhtml

were “real” or what the associated “risk” would be (see Table 3). Related to this concern, some participants felt that, if secondary findings were to be offered, return of results should only be done by genetic experts and/or via Genetics Departments. One participant stated that he felt it would be “completely unsafe” for non-genetic clinicians to offer such findings to patients. Some also felt that Genetics Departments already have referral pathways in place (e.g. cancer screening) and that this was another reason that secondary findings should only be offered by genetic clinicians, if at all.

Fifth, some participants were concerned about what their clinical recommendations for their patients would be, and how they would advise their patients, based on secondary findings. Linked to this, some participants were concerned about whether the ‘list’ of secondary findings would change and whether patients would be ‘re-tested’ if/when that did happen.

The final sub-theme that emerged, reflecting all of the concerns above, was that it would be more straightforward if secondary findings were not offered in clinical practice. Several participants stated that clinicians already offer clinical exome sequencing and that secondary findings are not offered as part of that clinical service. These participants felt that the same approach should be taken with clinical genome sequencing. See Table 3 for illustrative quotes.

3. Views on how genome sequencing should be delivered in clinical practice

Participants’ views on genome sequencing in clinical practice fell into three overarching themes.

3.1. Non-genetics clinicians can offer genome sequencing in clinical practice *as long as they have appropriate training and are supported by healthcare professionals with genetics expertise*

Participants felt that non-genetic clinicians should be able to offer genome sequencing to patients in their clinical practice but that adequate training and support from genetics teams were critical. One participant stated that ideally consent would be conducted by a genetic counsellor but that this isn’t practical (see Table 4 for illustrative quote). For many participants, appropriate training was key: although most felt that the consent process did not need to be conducted by a genetic expert, they also felt that anyone offering genome sequencing consent to patients should only do so after adequate training.

One participant emphasised that non-genetic clinicians can take responsibility for the consent process for genome sequencing “but there needs to be the appropriate support systems in place,” and another suggested that clinicians could consent patients but that patients might at least need the “option” of being able

For peer review only

to talk to and ask questions of a genetic counsellor, genetic nurse or other genetic specialist at the time of consent.

Many participants felt that non-genetic clinicians should be involved in the return of results to patients, but highlighted that they would need support from genetics departments / teams in interpreting and discussing complex findings, including secondary findings and more complex main findings. Notably, in this context, several clinicians spoke of existing working relationships between genetics and other specialty groups and the importance of working in multidisciplinary teams, e.g.

> *"they know we'll be around when the results come back if they're not clear cut and they need somebody to discuss it with because they do that all the time with other tests that they request… They know we're here to help with the interpretation of that, they just need to know enough to be able to offer the right genetic test to the right patient, at the right time."* (Participant 14, genetic clinician)

3.2. Views on training content

Participants made a number of recommendations about what training non-genetic clinicians will need in order to ensure they have adequate knowledge to receive informed consent for genome sequencing from patients. As well as having a knowledge of basic genetics, they would need to emphasise that genome sequencing is optional, manage patients' expectations regarding turnaround time for results, and admit if they don't know the answer to a patient's questions. Participants also felt that non-genetic clinicians need guidance regarding who should be offered genome sequencing. The recommendations for training content are summarised in Table 5.

3.3. Views on training delivery

In addition to the content of the training, participants expressed views about how training for non-genetic clinicians might best be delivered, with several suggesting that all doctors and nurses should be trained at medical or nursing school as part of the curriculum. Participants also suggested that online training was helpful for some types of learning (e.g. basic genetics) and that this should be interactive (e.g. with videos) rather than only static written information, but that other types of learning (e.g. counselling) needed to be in-person (e.g. shadowing). Several people felt that non-genetic clinicians would be most likely to attend half day or one-day training days or study days, rather than longer modules. Several participants also felt that such training should be mandatory (including regular and refresher training) and that there should be some kind of

For peer review only - http://bmjopen.bmj.com/site/about/guidelines.xhtml

"National Standard", "certificate" or "certification". Finally, several participants talked about the key role that genetic experts are already playing and will continue to play in training and "upskilling" their non-genetic colleagues, by working closely with them and supporting them, and via "buddying systems" and "genomics champions". See Table 6 for further details and additional illustrative quotes.

DISCUSSION

In this study we found that healthcare professionals with experience of delivering genome sequencing in the UK NHS as part of the 100,000 Genomes Project had mixed views regarding moving genome sequencing into clinical practice. Positive views were expressed regarding the potential for genome sequencing to improve diagnoses of rare diseases, contribute to the rare disease knowledge base, and lead to improved treatments in the future. However, some concerns were also expressed: participants' worries primarily centred around clinical departments having adequate time and expertise to analyse and interpret the sequence data, whether non-genetic clinicians are adequately prepared to disclose results to patients, and issues regarding secondary findings. Specifically regarding offering secondary findings from genome sequencing in clinical practice, perceived benefits included improved prediction and prevention of complex diseases, advancing research, and being responsive to what patients want. Concerns about secondary findings included lack of evidence regarding the possibly lower penetrance of variants identified incidentally, lack of evidence regarding whether it is clinically useful to return such results, how clinicians should advise patients to act based on the results, the potential for such findings to cause patients anxiety, and again that non-genetic clinicians do not have the necessary expertise to counsel patients about secondary findings. Overall, however, participants felt that non-genetic clinicians *will* be able to offer genome sequencing to patients in clinical practice, *as long as* they have adequate training and support from colleagues expert in genetics, particularly for more challenging cases and complex findings. They also offered specific recommendations for what that training and support should look like and how it should be delivered.

Our findings have implications for clinicians and policymakers concerned with the future delivery of genome sequencing in clinical practice. Our findings suggest that non-genetic clinicians may be able to support patients in their decision-making, receive consent and order genomic sequencing tests as long as they have

For peer review only

adequate training and support from genetic teams to help with interpretation of results and explanations to patients in complex cases. Clear protocols and processes for referral to genetics for patients to discuss complex main findings or if a secondary finding is identified are also needed. In addition to being offered as part of medical school and nursing training, education for non-genetic health professionals about genome sequencing technology (e.g. basic genetics, processes, limitations, and interpreting genetics reports) may be delivered online as long as it includes interactive multi-media components. Education about supporting consent and communicating results to patients may be better delivered through in-person training, and shadowing in the clinic may also be valuable. An important element of training in the future is that genetic experts need to play a key role in training and then supporting non-genetic clinicians in understanding sequence data and interpretation of results returned to them from the laboratories and results disclosure to patients.

Our finding that most participants felt health professionals from non-genetics backgrounds should be able to offer genome sequencing with support from genetics colleagues, perhaps reflects the increasing recognition that genetics/genomics is relevant to all areas of medicine and should therefore be 'mainstreamed'.[11-13] As others have concluded, it is inevitable that genomic sequencing will be used more frequently in mainstream clinical practice,[14] and that input from genetic professionals will be needed for more complex cases, in education and for the cross-discipline collaboration that will be essential for classifying variants and understanding phenotypes.[14] As the UK Genomic Medicine Service is established, it is important that research continues into how genome sequencing can best be offered, and how genetic and non-genetic professionals can work together to ensure patients with rare diseases have access to a Genomic Medicine Service that is effective and equitable.

Our findings regarding secondary findings suggest two possible ways forward. The first option is simply not to offer secondary findings in clinical practice, at least until further evidence regarding outcomes is obtained. As our participants stated, this would be consistent with how many clinical exomes are already managed today. However, as other participants stated, there are potential advantages to offering secondary findings, for example, this could provide much needed evidence on both the outcomes of variants identified incidentally, provided care is taken to ensure the long-term follow-up required to collect such research data. Thus, the second option is to provide the training and resources to be able to offer secondary findings in clinical practice and to encourage patients to consent to research alongside their clinical care. In the words of Les Biesecker

For peer review only

[15], "[a]s a field, we should take full advantage of all opportunities to study these variants by searching them out, returning them to patients and research participants, and studying their utility for predictive medicine."

Strengths of our study include that participants came from several hospitals and had a range of clinical backgrounds, and that all participants' perspectives were informed by actual personal experience of offering genome sequencing to patients within a healthcare service, albeit as part of a research study. These healthcare professionals were among the first to offer genome sequencing within the UK NHS, and so they have insights that go beyond attitudes expressed in previous studies which mostly occurred before genome sequencing was available and so participants did not have this real-world experience. In these earlier studies, participants' views were often extremely positive, including regarding secondary findings.[16-18] For example, in a 2013 US survey, 96% of clinical genetic healthcare professionals stated they believed secondary findings should be offered to adult patients.[16] The views expressed by our participants are more aligned with the more cautious views expressed by the genetic professionals, who also had experience of delivering genome sequencing, in a recent UK qualitative interview study focused on secondary findings.[9] These differences may, however, also reflect cultural differences between health professionals in the US and the UK. Weaknesses of our study include the small number of participants, meaning the findings may be potentially less generalisable to the wider population of healthcare professionals, and comparisons could not be made between genetic and non-genetic clinicians. In addition, the healthcare professionals were recruited from only two of the 13 Genomic Medicine Centres (GMCs) in the UK, and so the findings may not generalise to other geographic locations. The study was also conducted before most of the healthcare professionals had disclosed results to patients, so questions regarding potential benefits are still more hypothetical.

In conclusion, genetic and non-genetic healthcare professionals responsible for delivering clinical genome sequencing have largely positive views of genome sequencing in terms of improving diagnostic yield, but also have concerns and recommendations about practical aspects of delivery. Additional empirical research evidence will be useful to inform policy and practice, including how genome sequencing compares to exome sequencing and the clinical utility of secondary findings.

For peer review only

Competing interests statement

C.P. has been on a secondment with Genomics England as Clinical Lead for Genetic Counselling since October 2016. The other authors declare no conflicts of interest.

For peer review only

Authors contributions

SCS, MH and CL were responsible for data acquisition. SCS and MH were responsible for drafting the manuscript and data analysis. CL contributed to data analysis. CL and LSC conceived and designed the study, and contributed to interpretation of data. All authors contributed to revision of the manuscript and approve the final manuscript.

For peer review only

Acknowledgements

This manuscript presents independent research funded by the National Institute for Health Research (NIHR) under the Research for Patient Benefit funding stream (PB-PG-1014-35016: A study to define patient priorities and preferences when consenting to whole genome sequencing to ensure informed choice). The research was also funded by the NIHR Great Ormond Street Hospital (GOSH) Biomedical Research Centre (BRC). The research was made possible through access to patients being recruited to the 100,000 Genomes Project. which is managed by Genomics England Limited (a wholly owned company of the Department of Health) and is funded by the NIHR and NHS England. We also gratefully acknowledge the contribution of Andrew Buckton, clinical scientist, and Dominique Stephenson, patient representative, who both gave feedback on an early draft of the manuscript. LSC is partially funded by the NIHR GOSH BRC. The research funded is independent, and the views expressed are those of the authors and not necessarily those of the NHS, the NIHR or the Department of Health.

For peer review only

Table 1. Participant characteristics

Characteristic	N (%)
Gender	
Female	19 (82.6%)
Male	4 (17.4%)
Age	
20-30yrs	7 (30.4%)
31-40yrs	1 (4.3%)
41-50yrs	8 (34.8%)
51-60yrs	7 (30.4%)
Role	
Genetic clinician	5 (21.7%)
Non-genetic clinician	8 (34.8%)
Consenter	10 (43.5%)
Completed formal online training for 100,000 Genomes Project	
Yes	13 (56.5%)
No	10 (43.5%)

For peer review only

Table 2. Health professionals' attitudes (perceived benefits and concerns) towards moving genome sequencing into clinical practice

Content topic	Illustrative quote
**Perceived benefits**	
1. Improved diagnoses for patients	*"obviously we will get lots more diagnoses and that's good, I think that's good for everyone."* (Participant 13, genetic clinician)
2. Contributions to knowledge base	*"In terms of knowledge I think it's important. I'm not so sure it will be translated in to improvements in the quality of life for the patient."* (Participant 15, non-genetic clinician)
3. Improved treatments for future patients	*"…so if we can really correlate the clinical information with the genetic information and find these markers that will allow this personalised treatment, then we have a big step forward…."* (Participant 11, non-genetic clinician)
**Concerns**	
1. Lack of evidence	*I think it's too early to know whether or not it should become a routine part of clinical practice. I think we've now got sufficient evidence that whole exome sequencing can be very useful clinically… my own view is that we shouldn't roll out whole genome sequencing clinically until we have objective scientific evidence that it's superior to whole exome sequencing."* (Participant 14, genetic clinician)
2. Informed consent	*"…having overheard conversations from colleagues, I don't think they fully understand the limitations on what they are offering. I think clarity as to what this test will tell you, what it will not tell you. I think that needs to be clarified. And I think the colleagues need to understand before they offer it to their patients."* (Participant 12, genetic clinician)<br><br>*"What concerns me is that every single member of the Hundred Thousand Genomes team has said to me that it's an hour to do the full consent process… I think most clinicians don't have an hour spare to be going through that with patients… I think it's very difficult for clinicians to do that genetic counselling. I don't think it will be done particularly well, because it certainly won't, it will be a 5 minute process."* (Participant 17, non-genetic clinician)
3. Analysis and interpretation	*"presumably we would still have to do the phenotype data and certainly for clinicians, that's the limiting step, filling out another form!"* (Participant 23, non-genetics clinician)<br><br>*"It takes a lot of lab time to look at the data, it takes a lot of clinician time, to prepare the cases for a multidisciplinary team meeting..."* (Participant 13, genetic clinician)
4. Disclosing results	*"I think the problem is that other doctors think they're trained but I don't think they are… I'm much less confident about explanations of results… one sees all the time problems that arise because of that so results are over interpreted… so I am a bit worried about that kind of thing. Because I already see it…"* (Participant 13, genetic clinician)

For peer review only

Table 3. Health professionals' attitudes (perceived benefits and concerns) towards secondary findings

Content topic	Illustrative quote
**Perceived benefits**	
1. Improved prediction, prevention or early diagnosis of complex diseases for current patients	*"…and also I think it's part of offering, it's part of helping people to get healthier. If we can prevent things happening or help them earlier in the stage of something happening, I think it's part of our duty to actually do that."* (Participant 7, consenter)
2. Opportunity to advance research on the impact of secondary findings on patients	*"And as I said as we learn more we're going to appreciate number one how people receive that information and the impact it has on them, sort of medically as well as socially, financially et cetera…. So yeah I think it's just going to be a moving field and we're going to learn from mistakes, but you know probably gain some of the understanding."* (Participant 18, non-genetic clinician)
3. Being responsive to what patients want	*"Yeah I think you know, if there's something you can do about it, then I think that's fine yes. I think a lot of people would want to know and they can be consented up front."* (Participant 22, genetic clinician)
**Concerns**	
1. Evidence is lacking regarding the penetrance of variants identified incidentally	*"…the principle I think is sound but I think what we may find is that the penetrance of a lot of these things is not as high as we thought it was and that maybe if, if you ascertain somebody as a carrier of a pathogenic mutation completely incidentally what does that mean in terms of penetrance, I think we may be over, over estimating penetrance…"* (Participant 13, genetic clinician)
2. Evidence is lacking regarding whether it is clinically useful to return secondary findings	*"I think they [secondary findings] should be done when it's clinically necessary not just for the hell of it"* (Participant 4, consenter).
3. Potential psychological impact of the results	*"…even when you do find your aneurism and… you tell them that their risk of developing something is very low but they tend to walk around thinking they've got a time bomb in their head anyway and the same [is] going to apply with genetics. Incidental findings as well probably much more so in fact."* (Participant 19, non-genetic clinician)
4. Non-genetics clinicians are not prepared to discuss secondary findings with patients	*"…if we were to offer this for routine clinical practice, you know, in a neurological hospital how can we possibly decide whether, you know, a mutation in a cancer gene is a polymorphism or real or what's the risk you know? We cannot counsel patients on all these other things."* (Participant 21, non-genetic clinician)
5. Not clear what clinical recommendations for patients would be based on secondary findings	*"I don't know whether these people should be on screening or not… I'm just not quite sure we're there you see…"* (Participant 9, genetic clinician).
6. More straightforward if secondary findings were not offered as part of genome sequencing in clinical practice	*"Yeah well again I would not treat this as any way different than to the clinical exome we do here or the exome we do on research. You look at the things that you are interested in."* (Participant 11, non-genetic clinician)

For peer review only

Table 4. Health professionals' views on whether/how genome sequencing should be delivered by non-genetic clinicians in clinical practice

Content topic	Illustrative quote
**Non-genetic clinicians can offer genome sequencing in clinical practice *as long as they have appropriate training and are supported by healthcare professionals with genetics expertise***	
1. Not practical to have genetics experts (e.g. genetic counsellors) to offer & return all results	*"I mean in an ideal world actually what you'd do, what you would have is someone additional in your clinic perhaps a Genetic Counsellor who was working in the clinic who could do that kind of thing, but that's not how it works, it's not how we deploy our resources… I think it will be offered through other health professionals…"* (Participant 13, genetic clinician)
2. Non-genetic clinicians can offer genome sequencing as long as they have adequate training	*With appropriate training I think it probably could be offered by, by appropriate professionals…if it was a hospital Consultant and they had the time to really discuss it and they had the appropriate knowledge and training and the confidence to give the results back then I don't see that being a problem…"* (Participant 6, consenter)
3. Non-genetic clinicians can offer genome sequencing as long as they have sufficient support from genetics departments / teams in interpreting, returning and/or discussing complex findings	*"I think [offering genome sequencing] does need to be linked closely with genetics… I think that's key because otherwise you could potentially think something is relevant and pathogenic when actually it might not be or it's complicated with another variant… we must never underestimate the complexity of it… So I suppose that means that you would and should need access to your genetics team."* (Participant 23, non-genetic clinician)<br><br>*"So if you don't know anything about the genes then obviously the discussion of the findings should be left to somebody who has experience of genetics I do think yes."* (Participant 11, non-genetic clinician)

For peer review only

Table 5. Health professionals' views / recommendations for content of training for non-genetic clinicians

Content topic	Illustrative quote
Basic genetics	*"All doctors need to be trained in the basics of genetics"* (Participant 11, non-genetic clinician)
Guidance regarding who should be offered whole genome sequencing	*"I think we should, we need to have guidelines as to who should be the right candidate and as long as we have, we know that, then I think any professional would be able with the requisite training and who's informed about the indications, but […] then yes they should be able to offer it."* (Participant 15, non-genetic clinician)
Limitations / process / managing patients expectations	*"So I think it's essential that you understand the process, you understand the limitations, and you don't get patients, you have to give them honest expectations of process. And I think that's your own responsibility to try and understand the science of it. So yeah I think that's the most important thing not to give people false hope, not selling this as some magic new technology to give the answer to a problem. And the difficulty of interpretation and just why it takes so much time."* (Participant 18, non-genetic clinician)
Build confidence in ability to answer patients' questions	*"if you are not confident you cannot discuss genetic, because parents will ask questions. If you don't know the answer, then not going to work. You have to be confident when you have these discussions with parents I think."* (Participant 13, genetic clinician)
Admit if don't know answers to patients' questions	*"The offering it to them, if you're not sure of the answer tell them that you will find out, don't make it up."* (Participant 21, non-genetic clinician)
Emphasise patient has option to decline	*"I think what's happening also sometimes, when a consultant offers something to the patient, whatever it is, the patients are very keen to say yes because it's their specialist telling them something and rightly they think it's an important thing, but they don't always know that they have the right or the option to actually refuse it. So just to stress to the consultant that they have to make that option very clear. I think this option is not always as clear as it could be with consultant, so they should offer it but nothing more than that. […]This is a great test which I agree with but "You don't have to take part" usually is left out because we don't have time to do that. And I would love a little bit less of "Great" and a little bit more of "You have the option of not taking part"."* (Participant 7, consenter)
Allow adequate time to inform patients properly and to make their decision	*"it's important to know that… what we offer them has to be explained properly so they can make an informed consent and not using that to our advantage, just to get someone to consent into a study… What we're offering, we think it's this and that, and that, make it very clear, and let them make the informed consent and to take the decision in their time. Some people might need a little bit more time than others…"* (Participant 7, consenter)
How to understand / deal with a genetics report	*"How do you annotate a variant? What does it mean if it's a class one, two, three, four, five, and how do you interpret that? […] So I think these are things that just need to be clear to everybody how to understand a genetic report, what does make a class three, class four, whatever, but I think that's any clinician though."* (Participant 11, non-genetic clinician)

For peer review only

Table 6. Health professionals' views / recommendations for delivery of training for non-genetics clinicians

Training type	Illustrative quote
Medical school	*"Yeah. I think in the medical school."* (Participant 11, non-genetic clinician)
Nursing curriculum	*"Well I think it will be part of the curriculum in the future. It will just have to become part of nursing curriculum."* (Participant 8, consenter)
Online training	*"…the actual genetic concepts don't necessarily need to be in person they could be done with online modules"* (Participant 6, consenter)
Shadowing	HP: *Yeah, you shadow a consultant or a GC or someone before you get signed off to go and consent on your own…So when you do the online training, it gives you the knowledge but then when you're doing it practically, it gives you the ability to be able to speak to the patients, answer their questions as the PC doesn't speak to you, doesn't throw in little scenarios whereas in real life we know it doesn't go that straightforward, so doing it with someone and having somebody there actually works, it's what you need to do, yeah.* (Participant 10, consenter)
Role playing	*"I think it needs to be in person… the actual genetic concepts don't necessarily need to be in person they could be done with online modules, the talking to a patient the counselling aspect of it the understanding of the way that people deal with bad news and understand risk I think is better done in person. …the patient interacting side of it has to be done through face to face training…"* (Participant 6, consenter)
Day or half-day training	*"Yeah, I mean there are all sorts of courses available. The vast majority of clinicians haven't got time to do it and so they're more likely to come to a half day or a one day training course than they are to sit down and do some on line training. …although Genomics England has made available Masters and so on actually the sorts of people that are likely to want to request these in future haven't got the time to take a year or two out to do a Masters, they want something much more quick and practical and off the shelf and ready now, that just gets them up to speed so that they know enough knowledge to know which test to offer to whom and when."* (Participant 14, genetic clinician)<br><br>*"I guess an open training session would be useful, in terms of something like an FAQ sort of session as to what are the expected questions from families and you know how to [answer] them, that sort of a training would be helpful."* (Participant 15, non-genetic clinician)
Training should be mandatory / national Standard / certification	*"Mandatory is a way of keeping on top of who's actually been trained, when they were trained and you get your refresher so, yeah, you do your basic, you get your certificate and just things like that I think should be done, yeah."* (Participant 10, consenter)
Genetics clinicians train non-genetics colleagues / Buddying systems and genomics champions	*"So what we're trying to set up is a kind of a buddying system where we will try and have clinical genetics working with sort of genomics champion in that speciality…. So that the genomics champion from the speciality who will put themselves forward as a sacrificial lamb, and the genetics consultant or genetics counsellor with experience, will kind of discuss and agree what the steps are with feeding that information back to patients. … So what's happened is our genetics teams have gone to the cancer MDTs, disease specific cancer MDTs and they're trying to help people understand about the difference between somatic mutations and germ line mutations. And whether they are clinically actionable… And I think that is working quite well and it's quite labour intensive for the genetics team, but they are you know, working with particular oncologists who want to learn all about this. It's that kind of partnership between genetics and then somebody who's prepared to be the genomics champion from the speciality."* (Participant RC12, genetics clinician)

For peer review only

REFERENCES

1. Wright CF, FitzPatrick DR, Firth HV. Paediatric genomics: diagnosing rare disease in children. Nature reviews Genetics 2018;**19**(5):253-68 doi: 10.1038/nrg.2017.116[published Online First: Epub Date]|.
2. Bernhardt BA, Roche MI, Perry DL, et al. Experiences with obtaining informed consent for genomic sequencing. American journal of medical genetics Part A 2015;**167a**(11):2635-46 doi: 10.1002/ajmg.a.37256[published Online First: Epub Date]|.
3. Berrios C, James CA, Raraigh K, et al. Enrolling Genomics Research Participants through a Clinical Setting: the Impact of Existing Clinical Relationships on Informed Consent and Expectations for Return of Research Results. Journal of genetic counseling 2018;**27**(1):263-73 doi: 10.1007/s10897-017-0143-2[published Online First: Epub Date]|.
4. Tomlinson AN, Skinner D, Perry DL, et al. "Not Tied Up Neatly with a Bow": Professionals' Challenging Cases in Informed Consent for Genomic Sequencing. Journal of genetic counseling 2016;**25**(1):62-72 doi: 10.1007/s10897-015-9842-8[published Online First: Epub Date]|.
5. Walser SA, Werner-Lin A, Mueller R, et al. How do providers discuss the results of pediatric exome sequencing with families? Personalized medicine 2017;**14**(5):409-22 doi: 10.2217/pme-2017-0015[published Online First: Epub Date]|.
6. Wynn J, Lewis K, Amendola LM, et al. Clinical providers' experiences with returning results from genomic sequencing: an interview study. BMC Medical Genomics 2018;**11**(1):45 doi: 10.1186/s12920-018-0360-z[published Online First: Epub Date]|.
7. Pet DB, Holm IA, Williams JL, et al. Physicians' perspectives on receiving unsolicited genomic results. Genetics in Medicine 2018 doi: 10.1038/s41436-018-0047-z[published Online First: Epub Date]|.
8. Townsend A, Adam S, Birch PH, et al. "I want to know what's in Pandora's Box": comparing stakeholder perspectives on incidental findings in clinical whole genomic sequencing. American journal of medical genetics Part A 2012;**158a**(10):2519-25 doi: 10.1002/ajmg.a.35554[published Online First: Epub Date]|.
9. Ormondroyd E, Mackley MP, Blair E, et al. "Not pathogenic until proven otherwise": perspectives of UK clinical genomics professionals toward secondary findings in context of a Genomic Medicine Multidisciplinary Team and the 100,000 Genomes Project. Genetics in medicine : official journal of the American College of Medical Genetics 2018;**20**(3):320-28 doi: 10.1038/gim.2017.157[published Online First: Epub Date]|.
10. Braun VC, V. Using Thematic Analysis in Psychology. Qualitative Research in Psychology 2006;**3**:34
11. Bennett CL, Burke SE, Burton H, et al. A toolkit for incorporating genetics into mainstream medical services: Learning from service development pilots in England. BMC health services research 2010;**10**:125 doi: 10.1186/1472-6963-10-125[published Online First: Epub Date]|.
12. Burton H, Alberg C, Stewart A. Mainstreaming genetics: a comparative review of clinical services for inherited cardiovascular conditions in the UK. Public health genomics 2010;**13**(4):235-45 doi: 10.1159/000279625[published Online First: Epub Date]|.
13. Collins FS, Guttmacher AE. Genetics moves into the medical mainstream. Jama 2001;**286**(18):2322-4
14. Hammond CL, Willoughby JM, Parker MJ. Genomics for paediatricians: promises and pitfalls. Archives of disease in childhood 2018;**103**(9):895-900 doi: 10.1136/archdischild-2017-314558[published Online First: Epub Date]|.
15. Biesecker LG. Incidental variants are critical for genomics. American journal of human genetics 2013;**92**(5):648-51 doi: 10.1016/j.ajhg.2013.04.001[published Online First: Epub Date]|.
16. Lemke AA, Bick D, Dimmock D, et al. Perspectives of clinical genetics professionals toward genome sequencing and incidental findings: a survey study. Clin Genet 2013;**84**(3):230-6 doi: 10.1111/cge.12060[published Online First: Epub Date]|.
17. Middleton A, Morley KI, Bragin E, et al. Attitudes of nearly 7000 health professionals, genomic researchers and publics toward the return of incidental results from sequencing research. European journal of human genetics : EJHG 2016;**24**(1):21-9 doi: 10.1038/ejhg.2015.58[published Online First: Epub Date]|.

For peer review only - http://bmjopen.bmj.com/site/about/guidelines.xhtml

18. Yu JH, Harrell TM, Jamal SM, et al. Attitudes of genetics professionals toward the return of incidental results from exome and whole-genome sequencing. American journal of human genetics 2014;**95**(1):77-84 doi: 10.1016/j.ajhg.2014.06.004[published Online First: Epub Date]|.

For peer review only

Interview guide for healthcare professionals

Background of participant and 100KGP training

1. What is your professional background? e.g. cardiologist, paediatrician etc
2. Had you offered whole genome sequencing in either a research or clinical capacity prior to the 100,000 Genomes Project beginning?
3. Can you briefly describe your role in the 100,000 Genomes Project?
4. Prior to recruiting participants into the 100,000 Genomes Project, did you have to undergo any type of training? If so what training did you undergo?

Recruitment into the 100KGP

5. Which of your patients do you discuss the 100KGP with?
6. Can you describe the discussion you have with those patients?
   a. What do you tell them about the 100KGP?
   b. What questions do they ask about the 100KGP?
   c. How long does that discussion tend to last?
   d. Have there been any difficulties you have encountered whilst discussing the 100KGP with patients?
7. If the patient is interested in taking part in the 100KGP what do you do next?
8. Have you had many people that decline to be referred into the 100KGP?
   a. What are their reasons for declining?
9. Have you come up against any problems recruiting into the study e.g. organisational issues or other difficulties?

Return of results in the 100KGP

10. Have you returned any results from the 100KGP to your patients?

If participant HAS returned results

   a. Can you describe how you receive those results?
   b. What do you do once you have received those results? (write to patient? ask them to come to a clinic appointment?)

11. What kinds of results have you given to patients? (Primary findings; secondary findings)
12. What has the experience of returning results from WGS been like for you?
   a. Do you feel adequately prepared to discussion WGS results with patients?

For peer review only

b. Have you encountered any difficulties?

c. Are you comfortable returning secondary findings?

13. What are patients' reactions to receiving results?

a. What type of questions to patients ask?

b. What concerns if any do they have?

c. Do you feel able to address any concerns that they have?

If participant HAS NOT returned results

14. Do you know how the results will be sent to you?

a. How will you contact the patient?

b. Do you feel adequately prepared to discuss WGS results?

c. Are you comfortable with returning secondary findings?

d. Can you anticipate any difficulties?

Moving WGS into routine clinical practice

15. What do you think about whole genome sequencing being offered in routine clinical practice after the 100,000 Genomes Project has been completed?

a. What do you see as the main advantages, benefits?

b. What concerns, if any, do you have about offering whole genome sequencing being offered in routine clinical practice?

c. In your view, what (if any) are the main limitations of using whole genome sequencing for routine clinical practice?

d. In your view, what (if any) are the main challenges of offering whole genome sequencing in routine clinical practice?

16. Is there anything you would like to change about how whole genome sequencing is offered?

17. What advice would you give to other healthcare professionals discussing whole genome sequencing with patients?

18. What are your thoughts about how we would train health professionals that might be expected to offer whole genome sequencing in the future?

For peer review only

Interview guide for consenters

Background of participant and 100KGP training

19. What is your professional background? e.g. genetic counsellor, research fellow etc
20. What experience did you have of offering genetic testing or sequencing to patients prior to your involvement in the 100,000 Genomes Project?
    a. Specifically, had you offered whole genome sequencing prior to the 100,000 Genomes Project beginning?
21. Do you have any other relevant experience that you have brought to the role, e.g. consenting patients for research?
22. Can you briefly describe your role in the 100,000 Genomes Project?
23. Prior to recruiting participants into the 100,000 Genomes Project, what training did you undergo?
    a. Training specifically provided by the 100,000 Genomes Project
    b. Other training
24. What are your thoughts on the training that you did?
    a. Do you have any thoughts about the way it was delivered?
    b. Did you feel it adequately prepared you to recruit into the 100KGP?
    c. Did you feel anything was missing / could be improved?

Recruitment into the 100KGP

25. What has been your experience of recruiting patients into the 100KGP?
    a. What aspects of recruitment have worked well?
    b. What aspects of recruitment haven't worked well?
    c. How might recruitment be improved in your opinion?
26. Have you come up against any other problems recruiting into the study e.g. organisational issues or other difficulties?

Assessing informed consent in the 100KGP

27. What is your approach to consenting patients into the 100KGP?
    a. How do you generally begin the conversation?
    b. Are there set ways you conduct the session i.e. when you go through the consent form, family history etc or does it alter depending on the participants?

For peer review only

c. Does the discussion tend to be led by you or by the patient?

28. When you are talking to patients about consenting to the 100,000 Genomes Project, do you try to explore their understanding of the Project?

    a. If yes, how do you do this? Do you use any particular techniques to elicit patients' knowledge and understanding? What are they?

29. When you are talking to patients about consenting to the 100,000 Genomes Project, do you try to explore their attitudes, views, preferences, concerns around the Project?

    a. If yes, how do you do this? Do you use any particular techniques to elicit patients' attitudes and/or concerns? What are they?

30. What (if any) do you think are the main barriers to helping patients make informed choices about taking part in the 100,000 Genomes Project? (length of appointment; cultural barriers; lack of tools to support the consent discussion)

31. How does the method of contact i.e. whether they know about the 100KGP when they arrive at the hospital or whether they are recruited unexpectedly following another appointment, impact the consent discussion?

    a. Does it make a difference if they have already received written information about the study prior to the consent appointment?

Participants experiences in the 100KGP

32. What are the common questions patients have about taking part in the study?

    a. What concerns do patients have about taking part?

    b. What are their reasons for declining to take part?

33. Which are the main aspects of the study that you feel you spend most time discussing with patients (e.g. what genome sequencing is, secondary findings, accessing data etc)?

34. Do you think patients are putting a lot of thought into the decision to accept/decline testing? Do any want additional time to think about it?

35. What has been your experience discussing secondary findings with patients?

    a. Is it something that generates discussion and if so what kind of discussion?

    b. Who is the discussion between? (patient / parents themselves? Patient and health professional?)

36. Have you had experience of receiving consent or assent from a patient under the age of 16? What has been your experience?

37. Have you had to reconsent anyone turning 16 to remain in the project (with capacity)? What has been your experience of doing this?

    a. Has anyone withdrawn?

For peer review only

Moving WGS into routine clinical practice

38. What do you think about whole genome sequencing being offered in routine clinical practice after the 100,000 Genomes Project has been completed?

    a. What do you see as the main advantages, benefits?

    b. What concerns, if any, do you have about offering whole genome sequencing being offered in routine clinical practice?

    c. In your view, what (if any) are the main limitations of using whole genome sequencing for routine clinical practice?

    d. In your view, what (if any) are the main challenges of offering whole genome sequencing in routine clinical practice?

39. Is there anything you would like to change about how whole genome sequencing is offered?

40. What advice would you give to other healthcare professionals discussing whole genome sequencing with patients?

41. What are your thoughts about how we would train health professionals that might be expected to offer whole genome sequencing in the future?

For peer review only

Standards for Reporting Qualitative Research (SRQR)*

http://www.equator-network.org/reporting-guidelines/srqr/

Title and abstract

	Page/line no(s).
**Title** - Concise description of the nature and topic of the study Identifying the study as qualitative or indicating the approach (e.g., ethnography, grounded theory) or data collection methods (e.g., interview, focus group) is recommended	1
**Abstract** - Summary of key elements of the study using the abstract format of the intended publication; typically includes background, purpose, methods, results, and conclusions	2

Introduction

**Problem formulation** - Description and significance of the problem/phenomenon studied; review of relevant theory and empirical work; problem statement	3
**Purpose or research question** - Purpose of the study and specific objectives or questions	3

Methods

**Qualitative approach and research paradigm** - Qualitative approach (e.g., ethnography, grounded theory, case study, phenomenology, narrative research) and guiding theory if appropriate; identifying the research paradigm (e.g., postpositivist, constructivist/ interpretivist) is also recommended; rationale**	6
**Researcher characteristics and reflexivity** - Researchers' characteristics that may influence the research, including personal attributes, qualifications/experience, relationship with participants, assumptions, and/or presuppositions; potential or actual interaction between researchers' characteristics and the research questions, approach, methods, results, and/or transferability	6
**Context** - Setting/site and salient contextual factors; rationale**	5
**Sampling strategy** - How and why research participants, documents, or events were selected; criteria for deciding when no further sampling was necessary (e.g., sampling saturation); rationale**	5
**Ethical issues pertaining to human subjects** - Documentation of approval by an appropriate ethics review board and participant consent, or explanation for lack thereof; other confidentiality and data security issues	5
**Data collection methods** - Types of data collected; details of data collection procedures including (as appropriate) start and stop dates of data collection and analysis, iterative process, triangulation of sources/methods, and modification of procedures in response to evolving study findings; rationale**	5-6

For peer review only

**Data collection instruments and technologies** - Description of instruments (e.g., interview guides, questionnaires) and devices (e.g., audio recorders) used for data collection; if/how the instrument(s) changed over the course of the study	5-6
**Units of study** - Number and relevant characteristics of participants, documents, or events included in the study; level of participation (could be reported in results)	7
**Data processing** - Methods for processing data prior to and during analysis, including transcription, data entry, data management and security, verification of data integrity, data coding, and anonymization/de-identification of excerpts	6
**Data analysis** - Process by which inferences, themes, etc., were identified and developed, including the researchers involved in data analysis; usually references a specific paradigm or approach; rationale**	6
**Techniques to enhance trustworthiness** - Techniques to enhance trustworthiness and credibility of data analysis (e.g., member checking, audit trail, triangulation); rationale**	6

Results/findings

**Synthesis and interpretation** - Main findings (e.g., interpretations, inferences, and themes); might include development of a theory or model, or integration with prior research or theory	7-11
**Links to empirical data** - Evidence (e.g., quotes, field notes, text excerpts, photographs) to substantiate analytic findings	7-11 & Tables

Discussion

**Integration with prior work, implications, transferability, and contribution(s) to the field -** Short summary of main findings; explanation of how findings and conclusions connect to, support, elaborate on, or challenge conclusions of earlier scholarship; discussion of scope of application/generalizability; identification of unique contribution(s) to scholarship in a discipline or field	12
**Limitations** - Trustworthiness and limitations of findings	14

Other

**Conflicts of interest** - Potential sources of influence or perceived influence on study conduct and conclusions; how these were managed	15
**Funding** - Sources of funding and other support; role of funders in data collection, interpretation, and reporting	17

*The authors created the SRQR by searching the literature to identify guidelines, reporting standards, and critical appraisal criteria for qualitative research; reviewing the reference lists of retrieved sources; and contacting experts to gain feedback. The SRQR aims to improve the transparency of all aspects of qualitative research by providing clear standards for reporting qualitative research.

For peer review only - http://bmjopen.bmj.com/site/about/guidelines.xhtml

**The rationale should briefly discuss the justification for choosing that theory, approach, method, or technique rather than other options available, the assumptions and limitations implicit in those choices, and how those choices influence study conclusions and transferability. As appropriate, the rationale for several items might be discussed together.

Reference:

O'Brien BC, Harris IB, Beckman TJ, Reed DA, Cook DA. **Standards for reporting qualitative research: a synthesis of recommendations.** Academic Medicine, Vol. 89, No. 9 / Sept 2014 DOI: 10.1097/ACM.0000000000000388

For peer review only

BMJ Open

Delivering genome sequencing in clinical practice: an interview study with healthcare professionals involved in the 100,000 Genomes Project

Journal:	*BMJ Open*
Manuscript ID	bmjopen-2019-029699.R1
Article Type:	Original research
Date Submitted by the Author:	10-Jul-2019
Complete List of Authors:	Sanderson, Saskia; Great Ormond Street Hospital For Children NHS Foundation Trust, NE Thames Regional Genetics Service; University College London<br>Hill, Melissa; Great Ormond Street Hospital For Children NHS Foundation Trust, ; NE Thames Regional Genetics<br>Patch, Christine; King's College London<br>Searle, Beverly; Unique - Understanding Rare Chromosome and Genetic Disorders<br>Lewis, Celine; NE Thames Regional Genetics; UCL<br>Chitty, Lyn; NE Thames Regional Genetics; UCL, Institute of Child Health
**Primary Subject Heading**:	Genetics and genomics
Secondary Subject Heading:	Paediatrics, Genetics and genomics, Health policy
Keywords:	QUALITATIVE RESEARCH, genome sequencing, next-generation sequencing, MEDICAL EDUCATION & TRAINING, interviews

SCHOLARONE™
Manuscripts

Delivering genome sequencing in clinical practice: an interview study with healthcare professionals involved in the 100,000 Genomes Project

Saskia Sanderson[1,2], Melissa Hill[1],* Chris Patch[3,4,5], Beverly Searle[6], Celine Lewis[1],** Lyn Chitty[1]

*Joint first author

**Joint last author

[1] North East Thames Regional Genetics Service, Great Ormond Street Hospital for Children NHS Foundation Trust, London, UK

[2] Institute of Health Informatics, University College London, London, UK

[3] Florence Nightingale Faculty of Nursing and Midwifery, King's College London, London, UK

[4] Genomics England, Queen Mary University of London, Dawson Hall, London, UK

[5] Society and Ethics Research, Wellcome Genome Campus, Cambridge, UK

[6] Unique – Rare Chromosome & Gene Disorder Support Group, Oxted, UK

Key words: *genome sequencing, next-generation sequencing, medical education and training, qualitative research, interviews*

Corresponding author

Dr Melissa Hill

NE Thames Regional Genetics Service

Great Ormond Street Hospital

Level 5, Barclay House

37 Queen Square

London WC1N 3BH

Email: melissa.hill@ucl.ac.uk

For peer review only

ABSTRACT

Objectives: Genome sequencing is poised to be incorporated into clinical care for diagnoses of rare diseases and some cancers in many parts of the world. Healthcare professionals are key stakeholders in the clinical delivery of genome sequencing-based services. Our aim was to explore views of healthcare professionals with experience of offering genome sequencing via the 100,000 Genomes Project.

Design: Interview study using thematic analysis.

Setting: Four NHS hospitals in London.

Participants: Twenty-three healthcare professionals (5 genetic clinicians and 8 non-genetic clinicians (all Consultants), and 10 'consenters' from a range of backgrounds) involved in identifying or consenting patients for the 100,000 Genomes Project.

Results: Most participants expressed positive attitudes towards genome sequencing in terms of improved ability to diagnose rare diseases, but many also expressed concerns, with some believing its superiority over exome sequencing had not yet been demonstrated, or worrying that non-genetic clinicians are inadequately prepared to discuss genome sequencing results with patients. Several emphasised additional evidence about utility of genome sequencing in terms of both main and secondary findings is needed. Most felt non-genetic clinicians could support patients during consent, as long as they have appropriate training and support from genetic teams. Many stated genetics experts will play a vital role in training and supporting non-genetic clinicians in variant interpretation and results delivery, particularly for more complex cases.

Conclusions: Healthcare professionals responsible for delivering clinical genome sequencing have largely positive views the potential to improve diagnostic yield, but also significant concerns about practical aspects of offering these tests. Non-genetic clinicians delivering genome sequencing require guidance and support. Additional empirical evidence is needed to inform policy and practice, including: how genome compares to exome sequencing; utility of secondary findings; training, in particular of non-genetic health professionals; and mechanisms whereby genetics teams can offer appropriate support to their non-genetics colleagues.

For peer review only

STRENGTHS AND LIMITATIONS OF THIS STUDY

- The qualitative methodology employed in this study facilitated an in-depth exploration of interviewees' perspectives and allowed them to express their views in their own words; however, the sample size was small and encompassed only four London hospitals, which brings limitations for wider generalisability and did not allow between group comparisons
- Some of the interviewees were 'consenters' from the 100,000 Genomes Project so although they had real experience of supporting patients during the informed consent process, most will not be delivering genome sequencing clinically in the future
- Interviewees also included a range of genetic and non-genetic healthcare professionals from multiple hospital sites who *will* be responsible for delivering genome sequencing in the new UK Genomic Medicine Service
- Interviewees' views were informed by their experience of actually offering genome sequencing to NHS patients, rather than views being entirely based on hypothetical scenarios; however, at the time of the interviews very few results had been returned from the 100,000 Genomes Project
- Our findings shed important light on where the evidence gaps about clinical genome sequencing are from healthcare professionals' perspectives; this information may also be of use to practitioners and policymakers concerned with delivering genome sequencing clinically going forwards

For peer review only

BACKGROUND

Genome sequencing is increasingly being incorporated into clinical care for the purposes of diagnosing rare disease and some cancers. Genome sequencing allows us to determine most of a person's complete set of DNA by interrogating both the coding and non-coding regions of the genome. This contrasts with exome sequencing, which only looks at the coding regions of the genome or more targeted approaches that use gene panels to focus on specific sub-sets of candidate genes. Exome sequencing and panel testing have been widely used in both research and clinical settings, but as costs come down greater use of genome sequencing is occurring. Genome sequencing is more comprehensive than exome sequencing, with wider genome coverage and growing evidence of improved diagnostic yield in some contexts.[1] However, the potential for genomic sequencing to identify a wider range of variants as well as secondary findings that are unrelated to the indication for testing is a key consideration for implementation as this adds to the complexity of consent and return of results processes.

In the National Health Service (NHS) in England a new Genomic Medicine Service was launched in October 2018 that will include offering genome sequencing for patients with undiagnosed rare genetic diseases or a current diagnosis of cancer. A National Test Directory has been developed that describes the full range of tests available. The eligibility, testing and consent materials will be standardised across the Genomic Medicine Service.[2] The springboard for this new service has been the 100,000 Genomes Project which, under research consent and governance, recruited patients with rare diseases (children and adults) and their relatives, and patients with cancer, for genome sequencing between 2015 and 2018. Recruitment was conducted through thirteen NHS Genomic Medicine Centres located across the UK. In addition to receiving the main findings relevant to the indication for testing, participants could also opt in to secondary findings being looked for by the project scientists. The list of looked for secondary findings includes variants in genes that increase predisposition to breast and ovarian cancer (including *BRCA1* and *BRCA2*), Lynch syndrome (colorectal cancer and other cancers) and familial hypercholesterolemia. Participants could also opt in to secondary analysis of their genomic data to determine carrier status for cystic fibrosis. The return of main and secondary findings from the 100,000 Genomes Project is ongoing and participants are being given their results through NHS clinical pathways.

For peer review only

In England as elsewhere, there are efforts to mainstream genomic testing such that, when indicated, both genetic and non-genetic clinicians will be able to order genome sequencing for their patients, as they would other clinical tests. The main stated benefits of genome sequencing for rare disease diagnoses include increased discovery rate of causative gene variants, the potential to dramatically shorten the 'diagnostic odyssey', preventing multiple, often invasive and expensive, investigations and allowing targeted therapies.[3] However, moving genome sequencing from research to clinical practice raises many challenges. These include how to ensure there is the relevant expertise and infrastructure to deliver clinical genome sequencing nationally; whether non-genetic clinicians need support to offer genome sequencing and return results to patients as genomics is mainstreamed, and if so, what kind of support; and what results should be returned and to whom? While many of these questions arise in the context of other types of genetic tests, particularly exome sequencing, the potential challenges are even greater in the context of genome sequencing because of the sheer volume of data generated for any given individual or family.

Healthcare professionals are key stakeholders in answering these questions given they are central to offering genome sequencing to patients, facilitating informed consent, supporting patients' decision-making, and returning results to patients. Previous studies have explored healthcare professionals' experiences of obtaining informed consent for genomic sequencing[4-6] and returning genomic results,[7, 8] and views regarding secondary findings from genomic sequencing.[9, 10] Most of these studies were conducted in the US [4-9], one in Canada[10] and the only UK study to date drew on experiences of a single Multi-Disciplinary Team (MDT) and focused exclusively on secondary findings.[11]

The 100,000 Genomes Project affords a unique opportunity to gather stakeholder views on the implementation of genome sequencing in the setting of a large scale, nationally implemented initiative where healthcare is state funded and the intention is to return clinically relevant diagnoses. Here we aim to explore views of a range of healthcare professionals involved in the 100,000 Genomes Project regarding moving genome sequencing for rare disease diagnoses into clinical practice within the new Genomic Medicine Service, with a view to providing insights that may be relevant to policy and practice.

METHODS

Study design

For peer review only - http://bmjopen.bmj.com/site/about/guidelines.xhtml

A qualitative approach using one-to-one interviews for data collection was used to facilitate in-depth exploration of healthcare professionals' viewpoints on moving genome sequencing into NHS clinical practice.

Ethical approval

Ethical approval for this study was obtained from the West Midlands NHS Research Ethics Committee (15/WM/0258).

Patient and public involvement

The advisory team for this study had one lay-person and three patient support group representatives; including co-author BS, who has also contributed to the revision of the manuscript. Over the course of the study the advisory team has provided ongoing review and feedback on study conduct, research materials, interpretation of data and reporting of findings.

Sampling and recruitment

Participants were recruited from four London hospitals that were part of two Genomic Medicine Centres (GMCs) recruiting affected individuals and their relatives into the 100,000 Genomes Project. Three groups of health professionals were recruited to the study: (1) genetic and (2) non- genetic clinicians involved in identifying patients eligible to take part in the 100,000 Genomes Project; and (3) 'consenters', i.e. members of the 100,000 Genomes Project consenting teams whose role included consenting patients for genome sequencing. Potential participants in this study were identified by our research team and invited to participate via an email which included participant information explaining the aims of the study (see Supplemental Material). They were asked to contact the researchers (CL, MH, SS) if interested in participating. Written consent was obtained prior to the interviews to ensure informed and voluntary participation.

Participants were purposefully sampled across four London hospitals to ensure inclusion of participants from different geographical locations, various approaches to recruitment, and clinicians from a range of clinical backgrounds. Consenters in the 100,000 Genomes Project have a range of backgrounds, including genetic counselling, research, and other post graduate training. All consenters are trained, including taking the online course, "Preparing for the consent conversation" (https://www.genomicseducation.hee.nhs.uk/courses/courses/consent-ethics/). Interviews and analysis were undertaken concurrently, and recruitment ceased when no new themes were emerging during the interviews.

For peer review only - http://bmjopen.bmj.com/site/about/guidelines.xhtml

Interviews

An interview guide was developed for this study by the investigators to explore participants' experiences and views on (a) recruitment into and (b) return of results in the 100,000 Genomes Project, as well as their views on moving genome sequencing into clinical practice, and their professional background and training for the 100,000 Genomes Project. To develop the interview guide, a first draft of a set of questions reflecting the aims of the project was drafted by one of the investigators (MH) with experience in qualitative research methods; this was iteratively refined via discussion with two other investigators (CL, SS) on the team also with experience and expertise in qualitative methods; minor revisions were then made to the interview guide in response to questions that emerged during the first two interviews. There were two versions of the interview guide: one for clinicians and one for consenters (see Supplemental Material). The consenter version included additional sections about views on more specific aspects of informed consent and patients' experiences. The interviews were conducted face-to-face or via telephone by CL, MH or SS.

Data Analysis

Interviews were digitally recorded, transcribed verbatim and anonymised. Pseudonyms were then assigned to each participant. Data were analysed using the principles of thematic analysis.[12] NVivo version 10 (QSR International, Pty Ltd, Australia) software was used to facilitate coding and data analysis.

The focus of the analyses was text segments relevant to moving genome sequencing for rare disease diagnosis into clinical practice, reflecting the aims of this study. The research team comprised three postdoctoral researchers (SCS, CL, MH) with expertise in conducting qualitative analyses, two genetic health-care providers (CP, LSC) with experience supervising qualitative analyses, and one patient advocate (BS). The transcripts were coded by SCS and MH. The two investigators independently coded the same transcript and developed a draft codebook, and the two versions of the codebooks were compared and combined into a single codebook. The two investigators then independently coded a second transcript: a Kappa was calculated and indicated good inter-rater reliability. From this point the two investigators independently coded the remaining transcripts into eight broad overarching categories. The overarching category specifically focused on moving genome sequencing into routine clinical practice was then coded line by line into meaningful units of text. To ensure rigor and increase authenticity, multiple investigators (SCS, CL, MH) with varying levels of familiarity with genome sequencing checked the emerging themes at multiple time points and

For peer review only

together grouped the themes to form broader themes that were reviewed and redefined as the analysis progressed.

RESULTS

Participants

The 23 participants were 5 genetic clinicians (all Consultants in clinical genetics), 8 non-genetic clinicians (all Consultants, from a range of specialities including audiology, paediatric nephrology, neurology, and paediatrics), and 10 consenters (with a range of backgrounds, including nursing, project management, postdoctoral research, and medical doctor trainees) from four hospital sites. See Table 1 for additional participant characteristics. There were seven telephone and 16 face-to-face interviews. The interviews ranged in length from 19 to 75 minutes (median = 34 minutes).

Overarching themes

Participants' views on moving genome sequencing for rare disease diagnosis into clinical practice fell into three broad overarching themes as described below.

1. Attitudes towards moving genome sequencing into clinical practice

1.1. Perceived benefits

Participants talked about three main ways in which clinical genome sequencing will benefit patients. First, some participants felt that clinical genome sequencing will lead to improved diagnoses for patients with rare diseases by making the path to diagnosis more efficient, making it more likely patients will get a diagnosis with fewer tests needed. Second, some participants felt that clinical genome sequencing will add to the knowledge base about rare diseases. Third, several participants talked about how research will lead to improved treatments for future patients, either by improving disease progression stratification or by personalising treatments based on medication responsiveness (pharmacogenomics). See Table 2 for illustrative quotes.

1.2. Concerns

For peer review only

Although many participants expressed positive attitudes towards genome sequencing, some also raised concerns. First, a few participants expressed concern that genome sequencing has not been demonstrated to be superior to exome sequencing for diagnosing rare diseases, and believed genome sequencing should not be introduced clinically until there is scientific evidence about its value. Second, many participants had concerns about informed consent, particularly around the time needed for this. Participants emphasised that the consent form for the 100,000 Genomes Project was very long, and moreover that consent discussions had been undertaken by a dedicated consent team who would no longer be available after the transition to clinical practice. Some were concerned that some non-genetic clinicians do not understand the limitations of genome sequencing sufficiently, and that they are therefore under-prepared to offer genome sequencing to their patients, e.g.

> *"…having overheard conversations from colleagues, I don't think they fully understand the limitations on what they are offering. I think clarity as to what this test will tell you, what it will not tell you. I think that needs to be clarified. And I think the colleagues need to understand before they offer it to their patients."* (Participant 12, genetic clinician)

Third, participants voiced concerns about whether adequate resources – including laboratory and clinician time and expertise – will be available to analyse and interpret sequence data and subsequent variant validation, with several feeling genetic departments will be "swamped". Participants also raised concerns about phenotyping taking up the time of non-genetic clinicians, e.g.

> *"presumably we would still have to do the phenotype data and certainly for clinicians, that's the limiting step, filling out another form!"* (Participant 23, non-genetic clinician)

Concerns were also expressed about the potentially very long turn-around times to results. Several participants were also concerned that reports generated by labs were not "filtered" enough, meaning a lot of work was still left for clinicians to do. Fourth, a few participants expressed concerns about disclosing results to patients both in terms of the amount of consultation time needed to discuss results with patients and families, and whether non- genetic clinicians have insufficient understanding to be able to interpret and return more complex results. See Table 2 for additional illustrative quotes.

For peer review only - http://bmjopen.bmj.com/site/about/guidelines.xhtml

2. Attitudes towards offering secondary findings from genome sequencing

2.1. Perceived benefits

Participants perceived three main benefits of offering secondary findings from genome sequencing in clinical practice. First, several participants felt that offering clinically actionable secondary findings in the clinical setting will provide the opportunity to identify patients at increased disease risk, allowing for improved disease prediction, prevention or early diagnosis. Second, several participants felt that including secondary findings will lead to research learning opportunities: for example, how we will learn more about the medical, social and financial impact of secondary findings on patients. Third, several participants felt that patients want or would want to receive secondary findings, suggesting that offering this type of information might be responsive to patients' desires and preferences. See Table 3 for illustrative quotes.

2.2. Concerns

The issue of whether secondary findings should be offered to patients divided participants, with some advocating a cautious adoption whilst collecting data on penetrance and outcomes, and others stating that secondary findings should not be offered at all. First, some were concerned that evidence is lacking regarding the penetrance of variants identified "completely incidentally" and that we may be "over-estimating penetrance" as a consequence.

Related to this, the second sub-theme that emerged was a concern that evidence is lacking regarding whether it is "clinically useful" to return secondary findings to patients, particularly if they do not have a known family history of the associated condition. One participant said that this "opens a can of worms".

The third sub-theme that emerged was concern about the potential psychological impact of the results on participants, such as causing "anxiety". One participant asked, "How do you stop people from suffering unduly from getting these results back?" (Participant 8, consenter), while another asked, "are you opening up Pandora's box?" (Participant 5, consenter). One participant likened secondary findings to picking up aneurisms incidentally on brain scans (see Table 3).

The fourth sub-theme that emerged was a specific concern that non-genetic clinicians are not prepared to discuss secondary findings. One participant was concerned that doctors without "a genetics training background" might misinterpret secondary findings and that this could lead to "false reassurance" (Participant 6, consenter). Another emphasised that non-genetic clinicians couldn't decide whether mutations were "real" or what the associated disease "risk" would be (see Table 3). Related to this concern, some

For peer review only - http://bmjopen.bmj.com/site/about/guidelines.xhtml

participants felt that, if secondary findings were to be offered, return of results should only be done by genetic experts and/or via Genetics Departments. One participant stated that he felt it would be “completely unsafe” for non-genetic clinicians to offer such findings to patients. Some also felt that Genetics Departments already have referral pathways in place (e.g. cancer screening) and that this was another reason that secondary findings should only be offered by genetic clinicians, if at all.

Fifth, some participants were concerned about what their clinical recommendations for their patients would be, and how they would advise their patients, based on secondary findings. Linked to this, some participants were concerned about whether the ‘list’ of secondary findings would change and whether patients would be ‘re-tested’ if/when that did happen.

The final sub-theme that emerged, reflecting all of the concerns above, was that it would be more straightforward if secondary findings were not offered in clinical practice. Several participants stated that clinicians already offer clinical exome sequencing and that secondary findings are not offered as part of that clinical service in the UK. These participants felt that the same approach should be taken with clinical genome sequencing. See Table 3 for illustrative quotes.

3. Views on how genome sequencing should be delivered in clinical practice

Participants’ views on genome sequencing in clinical practice fell into three overarching themes.

3.1. Non-genetic clinicians can offer genome sequencing in clinical practice *as long as they have appropriate training and are supported by healthcare professionals with genetics expertise*

Participants felt that non-genetic clinicians should be able to offer genome sequencing to patients in their clinical practice but that adequate training and support from genetics teams were critical (Table 4). One participant stated that ideally consent would be conducted by a genetic counsellor but that this isn’t practical. For many participants, appropriate training was key: although most felt that the consent process did not need to be conducted by a genetic expert, they also felt that anyone offering genome sequencing consent to patients should only do so after adequate training.

One participant emphasised that non-genetic clinicians can take responsibility for the consent process for genome sequencing “but there needs to be the appropriate support systems in place,” and another suggested that clinicians could consent patients but that patients might at least need the “option” of being able to talk to and ask questions of a genetic counsellor, genetic nurse or other genetic specialist at the time of consent.

For peer review only

Many participants felt that non-genetic clinicians should be involved in the return of results to patients, but highlighted that they would need support from genetics departments / teams in interpreting and discussing complex findings, including secondary findings and more complex main findings. Notably, in this context, several clinicians spoke of existing working relationships between genetics and other specialty groups and the importance of working in multidisciplinary teams, e.g.

> *"they know we'll be around when the results come back if they're not clear cut and they need somebody to discuss it with because they do that all the time with other tests that they request… They know we're here to help with the interpretation of that, they just need to know enough to be able to offer the right genetic test to the right patient, at the right time."* (Participant 14, genetic clinician)

3.2. Views on training content

Participants made a number of recommendations about what training non-genetic clinicians will need in order to ensure they have adequate knowledge to receive informed consent for genome sequencing from patients. As well as having a knowledge of basic genetics, they would need to emphasise that genome sequencing is optional, manage patients' expectations regarding turnaround time for results, and admit if they don't know the answer to a patient's questions. Participants also felt that non-genetic clinicians need guidance regarding who should be offered genome sequencing. The recommendations for training content are summarised in Table 5.

3.3. Views on training delivery

In addition to the content of the training, participants expressed views about how training for non-genetic clinicians might best be delivered, with several suggesting that all doctors and nurses should be trained at medical or nursing school as part of the curriculum. Participants also suggested that online training was helpful for some types of learning (e.g. basic genetics) and that this should be interactive (e.g. with videos) rather than only static written information, but that other types of learning (e.g. counselling) needed to be in-person (e.g. shadowing). Several people felt that non-genetic clinicians would be most likely to attend half day or one-day training days or study days, rather than longer modules. Several participants also felt that such training should be mandatory (including regular and refresher training) and that there should be some kind of "National Standard", "certificate" or "certification". Finally, several participants talked about the key role that genetic experts are already playing and will continue to play in training and "upskilling" their non-genetic colleagues,

For peer review only

by working closely with them and supporting them, and via “buddying systems” and “genomics champions”. See Table 6 for further details and additional illustrative quotes.

DISCUSSION

In this study we found that healthcare professionals with experience of delivering genome sequencing in the UK NHS as part of the 100,000 Genomes Project had mixed views regarding moving genome sequencing into clinical practice. Positive views were expressed regarding the potential for genome sequencing to improve diagnoses of rare diseases, contribute to the rare disease knowledge base, and lead to improved treatments in the future. However, some concerns were also expressed. Some participants’ worries were primarily about whether healthcare professionals in genetics and non-genetics departments have adequate resources to explore the tiered variant report that is returned, undertake variant interpretation, conduct additional phenotyping and technically validate results to issue a report. They also had reservations as to whether non-genetic clinicians are adequately prepared to disclose results to patients. Similar concerns about whether clinicians, particularly non-genetic clinicians, have the necessary resources and training to include genome sequencing in their practice have been reported in a US study exploring health professional experiences of returning genome sequencing results.[8] Participants in the present study were also concerned about secondary findings from genome sequencing being offered in clinical practice. Although perceived benefits of offering secondary findings that included improved prediction and prevention of complex diseases, advancing research, and being responsive to what patients want were discussed, a number of concerns were also voiced. Concerns about reporting secondary findings to patients included lack of evidence regarding the possibly lower penetrance of variants identified incidentally, lack of evidence regarding whether it is clinically useful to return such results, how clinicians should advise patients to act based on the results, the potential for such findings to cause patients anxiety, and again that non-genetic clinicians do not have the necessary expertise to counsel patients about secondary findings. Overall, however, participants felt that non-genetic clinicians *will* be able to offer genome sequencing to patients in clinical practice, *as long as* they have adequate training and support from colleagues with expertise in genetics, particularly for more challenging cases and complex findings. They also offered specific recommendations for what that training and support should look like and how it should be delivered.

Our findings have implications for clinicians and policymakers concerned with moving genome sequencing into clinical practice. Our findings suggest that non-genetic clinicians will be able to support patients in their

For peer review only - http://bmjopen.bmj.com/site/about/guidelines.xhtml

decision-making, receive consent and order genomic sequencing tests as long as they have adequate training and support from genetic teams to help with interpretation of results and explanations to patients in complex cases. Clear protocols and processes for referral to genetics for patients to discuss complex main findings or if a secondary finding is identified are also needed. In addition to being offered as part of medical school and nursing training, education for non-genetic health professionals about genome sequencing technology (e.g. basic genetics, processes, limitations, and interpreting genetics reports) may be delivered online as long as it includes interactive multi-media components. Education about supporting consent and communicating results to patients may be better delivered through in-person training, and shadowing in the clinic may also be valuable. An important element of training in the future is that genetic experts need to play a key role in training and then supporting non-genetic clinicians in understanding sequence data and interpretation of results returned to them from the laboratories and results disclosure to patients.

Our finding that most participants felt health professionals from non-genetic backgrounds should be able to offer genome sequencing with support from genetics colleagues, perhaps reflects the increasing recognition that genetics/genomics is relevant to all areas of medicine and should therefore be 'mainstreamed'.[13-15] As others have concluded, it is inevitable that genomic sequencing will be used more frequently in mainstream clinical practice,[16] and that input from genetic professionals will be needed for more complex cases, in education and for the cross-discipline collaboration that will be essential for classifying variants and understanding phenotypes.[8, 16] As the UK Genomic Medicine Service is established, it is important that research continues into how genome sequencing can best be offered, and how genetic and non-genetic professionals can work together to ensure patients with rare diseases have access to a Genomic Medicine Service that is effective and equitable.

Approaches to the return of secondary findings from genomic sequencing in both research and clinical settings have varied widely, with some programmes choosing to report from a long list of secondary findings, and others opting not to offer any secondary findings at all. Professional guidelines for practice also differ. The American College of Medical Genetics (ACMG) recommends that all laboratories conducting clinical genome or exome sequencing for patients should search for DNA variants that are classified as pathogenic in a minimum list of 59 medically actionably genes, and that these secondary findings should be reported to patients, regardless of the original reason for the sequencing being done.[17] Canadian[18] and European guidelines[19] have taken a more conservative approach, and do not recommend secondary findings are looked

For peer review only

for in the clinical context at the present time. In the 100,000 Genomes Project, participants could opt to have the project scientists look for secondary findings in genes on a more limited list than the ACMG guidelines list (at the time of our interviews no secondary findings had been reported to clinicians or patients). The views of our participants regarding secondary findings suggest two possible ways forward. The first option is simply not to offer secondary findings in clinical practice, at least not until further evidence regarding outcomes is obtained. As some of our participants stated, this would be consistent with how many clinical exomes are already managed today. However, as other participants stated, there are potential advantages to offering secondary findings, for example, this could provide much needed evidence on both the outcomes of variants identified incidentally, provided care is taken to ensure the long-term follow-up required to collect such research data. Thus, the second option is to provide the training and resources to be able to offer secondary findings in clinical practice and to encourage patients to consent to research alongside their clinical care. In the words of Les Biesecker[20], *"[a]s a field, we should take full advantage of all opportunities to study these variants by searching them out, returning them to patients and research participants, and studying their utility for predictive medicine."*

Strengths of our study include that participants came from several hospitals and had a range of clinical backgrounds, and that all participants' perspectives were informed by actual personal experience of offering genome sequencing to patients within a healthcare service, albeit as part of a research study. These healthcare professionals were among the first to offer genome sequencing within the UK NHS, and so they have insights that go beyond attitudes expressed in previous studies which mostly occurred before genome sequencing was available and so participants did not have this real-world experience.[21] In some earlier studies, participants' views were often extremely positive, including regarding secondary findings.[22, 23] For example, in a 2013 US survey, 96% of clinical genetic healthcare professionals stated they believed secondary findings should be offered to adult patients.[22] The views expressed by our participants are more aligned with the more cautious views expressed by the genetic professionals, who also had experience of delivering genome sequencing where secondary findings were offered, in a recent UK qualitative interview study focused on secondary findings.[11] These differences may, however, also reflect overarching differences in the approach to reporting secondary findings between health professionals in the UK and the US, where many of the studies to date have been conducted, as the ACMG has advocated the reporting of a wide range of secondary findings from clinical genome sequencing since 2013.

For peer review only - http://bmjopen.bmj.com/site/about/guidelines.xhtml

Weaknesses of our study include the small number of participants, meaning the findings may be potentially less generalisable to the wider population of healthcare professionals, and comparisons could not be made between genetic and non-genetic clinicians. In addition, the healthcare professionals were recruited from only two of the 13 Genomic Medicine Centres (GMCs) in the UK, and so the findings may not generalise to other geographic locations. The study was also conducted before most of the healthcare professionals had disclosed results to patients, so questions regarding potential benefits are still more hypothetical.

In conclusion, genetic and non-genetic healthcare professionals responsible for delivering clinical genome sequencing in the UK have largely positive views of genome sequencing in terms of improving diagnostic yield, but also have concerns and recommendations about practical aspects of delivery. Additional empirical research evidence will be useful to inform policy and practice, including how genome sequencing compares to exome sequencing and the clinical utility of secondary findings.

REFERENCES

1. Bick D, Jones M, Taylor SL, et al. Case for genome sequencing in infants and children with rare, undiagnosed or genetic diseases. *Journal Med Genet* 2019; Apr 25 [Epub ahead of print]

2. Barwell JG, O'Sullivan RBG, Mansbridge LK, et al. Challenges in implementing genomic medicine: the 100,000 Genomes Project *J Transl Genet Genom* 2018;**2**:13.

3. Wright CF, FitzPatrick DR, Firth HV. Paediatric genomics: diagnosing rare disease in children. *Nat Rev Genet* 2018;**19**:253-68.

4. Bernhardt BA, Roche MI, Perry DL, et al. Experiences with obtaining informed consent for genomic sequencing. *Am J Med Genet Part A* 2015;**167a**:2635-46.

5. Berrios C, James CA, Raraigh K, et al. Enrolling genomics research participants through a clinical setting: the impact of existing clinical relationships on informed consent and expectations for return of research results. *J Genet Couns* 2018;**27**:263-73.

6. Tomlinson AN, Skinner D, Perry DL, et al. "Not tied up neatly with a bow": professionals' challenging cases in informed consent for genomic sequencing. *J Genet Couns* 2016;**25**:62-72.

7. Walser SA, Werner-Lin A, Mueller R, et al. How do providers discuss the results of pediatric exome sequencing with families? *Per Med* 2017;**14**:409-22.

8. Wynn J, Lewis K, Amendola LM, et al. Clinical providers' experiences with returning results from genomic sequencing: an interview study. *BMC Med Genomics* 2018;**11**:45.

For peer review only

9. Pet DB, Holm IA, Williams JL, et al. Physicians' perspectives on receiving unsolicited genomic results. *Genet Med* 2019;**21**:311-8.

10. Townsend A, Adam S, Birch PH, et al. "I want to know what's in Pandora's Box": comparing stakeholder perspectives on incidental findings in clinical whole genomic sequencing. *Am J Med Genet Part A*. 2012;**158a**:2519-25.

11. Ormondroyd E, Mackley MP, Blair E, et al. "Not pathogenic until proven otherwise": perspectives of UK clinical genomics professionals toward secondary findings in context of a Genomic Medicine Multidisciplinary Team and the 100,000 Genomes Project. *Genet Med* 2018;**20**:320-8.

12. Braun V, Clarke V. Using thematic analysis in psychology. *Qual Res Psychol* 2006;**3**:77-101.

13. Bennett CL, Burke SE, Burton H, et al. A toolkit for incorporating genetics into mainstream medical services: Learning from service development pilots in England. *BMC Health Serv Res* 2010;**10**:125.

14. Burton H, Alberg C, Stewart A. Mainstreaming genetics: a comparative review of clinical services for inherited cardiovascular conditions in the UK. *Public Health Genom* 2010;**13**:235-45.

15. Collins FS, Guttmacher AE. Genetics moves into the medical mainstream. *JAMA* 2001;**286**:2322-4.

16. Hammond CL, Willoughby JM, Parker MJ. Genomics for paediatricians: promises and pitfalls. *Arch Dis Child* 2018;**103**:895-900.

17. Kalia SS, Adelman K, Bale SJ, et al. Recommendations for reporting of secondary findings in clinical exome and genome sequencing, 2016 update (ACMG SF v2.0): a policy statement of the American College of Medical Genetics and Genomics. *Genet Med* 2017;**19**:249-55.

18. Boycott K, Hartley T, Adam S, et al. The clinical application of genome-wide sequencing for monogenic diseases in Canada: Position Statement of the Canadian College of Medical Geneticists. *J Med Genet* 2015;**52**:431-7.

19. van El CG, Cornel MC, Borry P, et al. Whole-genome sequencing in health care. Recommendations of the European Society of Human Genetics. *EJHG* 2013;**21 Suppl 1**:S1-5.

20. Biesecker LG. Incidental variants are critical for genomics. *Am J Hum Genet* 2013;**92**:648-51.

21. Mackley MP, Fletcher B, Parker M, et al. Stakeholder views on secondary findings in whole-genome and whole-exome sequencing: a systematic review of quantitative and qualitative studies. *Genet Med* 2017;**19**:283-93.

22. Lemke AA, Bick D, Dimmock D, et al. Perspectives of clinical genetics professionals toward genome sequencing and incidental findings: a survey study. *Clin Gen* 2013;**84**:230-6.

For peer review only

23. Yu JH, Harrell TM, Jamal SM, et al. Attitudes of genetics professionals toward the return of incidental results from exome and whole-genome sequencing. *Am J Hum Genet* 2014;**95**:77-84.

Data availability statement

The data from this study (deidentified interview transcripts) are available from the corresponding author upon reasonable request.

Competing interests statement

C.P. has been on a secondment with Genomics England as Clinical Lead for Genetic Counselling since October 2016. The other authors declare no conflicts of interest.

Authors contributions

SCS, MH and CL were responsible for data acquisition. SCS and MH were responsible for drafting the manuscript and data analysis. CL contributed to data analysis. CL and LSC conceived and designed the study, and contributed to interpretation of data. BS and CP were members of the advisory team who provided feedback on study design, research materials and findings. All authors contributed to revision of the manuscript and approve the final manuscript.

Acknowledgements

This manuscript presents independent research funded by the National Institute for Health Research (NIHR) under the Research for Patient Benefit funding stream (PB-PG-1014-35016: A study to define patient priorities and preferences when consenting to whole genome sequencing to ensure informed choice). The research was also funded by the NIHR Great Ormond Street Hospital (GOSH) Biomedical Research Centre (BRC). The research was made possible through access to patients being recruited to the 100,000 Genomes Project, which is managed by Genomics England Limited (a wholly owned company of the Department of Health) and is funded by the NIHR and NHS England. We also gratefully acknowledge the contribution of Andrew Buckton, clinical scientist, and Dominique Stephenson, patient representative, who both gave feedback on an early draft of the manuscript. LSC is partially funded by the NIHR GOSH BRC. The research funded is independent, and the views expressed are those of the authors and not necessarily those of the NHS, the NIHR or the Department of Health.

For peer review only

For peer review only

Table 1. Participant characteristics

Characteristic	N (%)
Gender	
Female	19 (82.6%)
Male	4 (17.4%)
Age	
20-30yrs	7 (30.4%)
31-40yrs	1 (4.3%)
41-50yrs	8 (34.8%)
51-60yrs	7 (30.4%)
Role	
Genetic clinician	5 (21.7%)
Non-genetic clinician	8 (34.8%)
Audiovestibular medicine	*2*
Neurology	*2*
Neuromuscular	*2*
Nephrology	*2*
Consenter	10 (43.5%)
Nursing	*3*
Project management	*1*
Laboratory research scientists	*2*
Medical doctor trainees	*1*
Other	*3*
Completed formal online training for 100,000 Genomes Project	
Yes	13 (56.5%)
No	10 (43.5%)

For peer review only

Table 2. Health professionals' attitudes (perceived benefits and concerns) towards moving genome sequencing into clinical practice

Content topic	Illustrative quote
**Perceived benefits**	
1. Improved diagnoses for patients	*"obviously we will get lots more diagnoses and that's good, I think that's good for everyone… and I think it helps, it helps for counselling, prevention, this type of things"* (Participant 16, non-genetic clinician)
2. Contributions to knowledge base	*"It's a technology that gives us access to, you know, vast quantities of information and if we as clinicians and scientists are able to interpret that in a meaningful way... and the more information we gather, then the easier it should be to interpret because we've got so many comparisons and so much more data to base our decisions on."* (Participant 18, non-genetic clinician)
3. Improved treatments for future patients	*"…so if we can really correlate the clinical information with the genetic information and find these markers that will allow this personalised treatment, then we have a big step forward…."* (Participant 11, non-genetic clinician)
**Concerns**	
1. Lack of evidence	*"I think it's too early to know whether or not [whole genome sequencing] should become a routine part of clinical practice. I think we've now got sufficient evidence that whole exome sequencing can be very useful clinically… my own view is that we shouldn't roll out whole genome sequencing clinically until we have objective scientific evidence that it's superior to whole exome sequencing."* (Participant 14, genetic clinician)
2. Informed consent	*"What concerns me is that every single member of the 100,000 genomes team has said to me that it's an hour to do the full consent process… I think most clinicians don't have an hour spare to be going through that with patients… I think it's very difficult for clinicians to do that genetic counselling. I don't think it will be done particularly well, because it certainly won't, it will be a 5 minute process."* (Participant 17, non-genetic clinician)
3. Resources for analysis and interpretation	*"It takes a lot of lab time to look at the data, it takes a lot of clinician time, to prepare the cases for a multidisciplinary team meeting..."* (Participant 13, genetic clinician)
4. Interpretation and disclosure of results	*"I think the problem is that other doctors think they're trained but I don't think they are… I'm much less confident about explanations of results… one sees all the time problems that arise because of that so results are over interpreted… so I am a bit worried about that kind of thing. Because I already see it…"* (Participant 13, genetic clinician)

For peer review only

Table 3. Health professionals' attitudes (perceived benefits and concerns) towards secondary findings

Content topic	Illustrative quote
**Perceived benefits**	
1. Improved prediction, prevention or early diagnosis of complex diseases for current patients	*"…and also I think it's part of offering, it's part of helping people to get healthier. If we can prevent things happening or help them earlier in the stage of something happening, I think it's part of our duty to actually do that."* (Participant 7, consenter)
2. Opportunity to advance research	*"And as I said as we learn more we're going to appreciate number one how people receive that information and the impact it has on them, sort of medically as well as socially, financially et cetera…. So yeah I think it's just going to be a moving field and we're going to learn from mistakes, but you know probably gain some of the understanding."* (Participant 18, non-genetic clinician)
3. Being responsive to what patients want	*"Yeah I think you know, if there's something you can do about it, then I think that's fine yes. I think a lot of people would want to know and they can be consented up front."* (Participant 22, genetic clinician)
**Concerns**	
1. Evidence is lacking regarding the penetrance of variants identified incidentally	*"…the principle I think is sound but I think what we may find is that the penetrance of a lot of these things is not as high as we thought it was and that maybe if, if you ascertain somebody as a carrier of a pathogenic mutation completely incidentally what does that mean in terms of penetrance, I think we may be over, over estimating penetrance…"* (Participant 13, genetic clinician)
2. Evidence is lacking regarding whether it is clinically useful to return secondary findings	*"I think they [secondary findings] should be done when it's clinically necessary not just for the hell of it"* (Participant 4, consenter).
3. Potential psychological impact of the results	*"…even when you do find your aneurism and… you tell them that their risk of developing something is very low but they tend to walk around thinking they've got a time bomb in their head anyway and the same [is] going to apply with genetics. Incidental findings as well probably much more so in fact."* (Participant 19, non-genetic clinician)
4. Non-genetic clinicians are not prepared to discuss secondary findings with patients	*"…if we were to offer this for routine clinical practice, you know, in a neurological hospital how can we possibly decide whether, you know, a mutation in a cancer gene is a polymorphism or real or what's the risk you know? We cannot counsel patients on all these other things."* (Participant 21, non-genetic clinician)
5. Not clear what clinical recommendations for patients would be based on some secondary findings	*"I think in BRCA and the MMR gene there's plenty of evidence out there. So you know you'd have to have [screening], so it needs to be recurrent mutations that are definitely associated and so what do you do then if you go and find something else. I don't know whether these people should be on screening or not "* (Participant 9, genetic clinician)
6. More straightforward if secondary findings were not offered as part of genome sequencing in clinical practice	*"Yeah well again I would not treat this as any way different than to the clinical exome we do here or the exome we do on research. You look at the things that you are interested in."* (Participant 11, non-genetic clinician)

For peer review only

Table 4. Health professionals' views on whether/how genome sequencing should be delivered by non-genetic clinicians in clinical practice

Content topic	Illustrative quote
**Non-genetic clinicians can offer genome sequencing in clinical practice *as long as they have appropriate training and are supported by healthcare professionals with genetics expertise***	
1. Not practical to have genetics experts (e.g. genetic counsellors) to offer & return all results	*"I mean in an ideal world actually what you'd do, what you would have is someone additional in your clinic perhaps a Genetic Counsellor who was working in the clinic who could do that kind of thing, but that's not how it works, it's not how we deploy our resources… I think it will be offered through other health professionals…"* (Participant 13, genetic clinician)
2. Non-genetic clinicians can offer genome sequencing as long as they have adequate training	*"With appropriate training I think it probably could be offered by, by appropriate professionals…if it was a hospital Consultant and they had the time to really discuss it and they had the appropriate knowledge and training and the confidence to give the results back then I don't see that being a problem…"* (Participant 6, consenter)
3. Non-genetic clinicians can offer genome sequencing as long as they have sufficient support from genetic departments / teams in interpreting, returning and/or discussing complex findings	*"I think [offering genome sequencing] does need to be linked closely with genetics… I think that's key because otherwise you could potentially think something is relevant and pathogenic when actually it might not be or it's complicated with another variant… we must never underestimate the complexity of it… So I suppose that means that you would and should need access to your genetics team."* (Participant 23, non-genetic clinician)<br><br>*"So if you don't know anything about the genes then obviously the discussion of the findings should be left to somebody who has experience of genetics I do think yes."* (Participant 11, non-genetic clinician)

For peer review only

Table 5. Health professionals' views / recommendations for content of training for non-genetic clinicians

Content topic	Illustrative quote
Basic genetics	*"All doctors need to be trained in the basics of genetics"* (Participant 11, non-genetic clinician)
Guidance regarding who should be offered whole genome sequencing	*"I think we should, we need to have guidelines as to who should be the right candidate and as long as we have, we know that, then I think any professional would be able with the requisite training and who's informed about the indications, but […] then yes they should be able to offer it."* (Participant 15, non-genetic clinician)
Limitations / process / managing patients expectations	*"So I think it's essential that you understand the process, you understand the limitations… you have to give them honest expectations of process. And I think that's your own responsibility to try and understand the science of it. So yeah I think that's the most important thing not to give people false hope, not selling this as some magic new technology to give the answer to a problem. And the difficulty of interpretation and just why it takes so much time."* (Participant 18, non-genetic clinician)
Build confidence in ability to answer patients' questions	*"if you are not confident you cannot discuss genetic, because parents will ask questions. If you don't know the answer, then not going to work. You have to be confident when you have these discussions with parents I think."* (Participant 13, genetic clinician)
Admit if don't know answers to patients' questions	*"The offering it to them, if you're not sure of the answer tell them that you will find out, don't make it up."* (Participant 21, non-genetic clinician)
Emphasise patient has option to decline	*"I think what's happening also sometimes, when a consultant offers something to the patient, whatever it is, the patients are very keen to say yes because it's their specialist telling them something and rightly they think it's an important thing, but they don't always know that they have the right or the option to actually refuse it. So just to stress to the consultant that they have to make that option very clear. This is a great test which I agree with but "You don't have to take part" usually is left out because we don't have time to do that. And I would love a little bit less of "Great" and a little bit more of "You have the option of not taking part"."* (Participant 7, consenter)
Allow adequate time to inform patients properly and to make their decision	*"what we offer them has to be explained properly so they can make an informed consent and not using that to our advantage, just to get someone to consent into a study… What we're offering, we think it's this and that, and that, make it very clear, and let them make the informed consent and to take the decision in their time. Some people might need a little bit more time than others…"* (Participant 7, consenter)
How to understand / deal with a genetics report	*"How do you annotate a variant? What does it mean if it's a class one, two, three, four, five, and how do you interpret that? […] So I think these are things that just need to be clear to everybody how to understand a genetic report, what does make a class three, class four, whatever, but I think that's any clinician though."* (Participant 11, non-genetic clinician)

For peer review only

Table 6. Health professionals' views / recommendations for training for non-genetic clinicians

Training type	Illustrative quote
Medical school	*"Yeah. I think in the medical school."* (Participant 11, non-genetic clinician)
Nursing curriculum	*"Well I think it will be part of the curriculum in the future. It will just have to become part of nursing curriculum."* (Participant 8, consenter)
Online training	*"…the actual genetic concepts don't necessarily need to be in person they could be done with online modules"* (Participant 6, consenter)
Shadowing	"*you shadow a consultant or a genetic counsellor or someone before you get signed off to go and consent on your own…So when you do the online training, it gives you the knowledge but then when you're doing it practically, it gives you the ability to be able to speak to the patients, answer their questions as the PC doesn't speak to you, doesn't throw in little scenarios whereas in real life we know it doesn't go that straightforward, so doing it with someone and having somebody there actually works"* (Participant 10, consenter)
Role playing	*"talking to a patient, the counselling aspect of it, the understanding of the way that people deal with bad news and understand risk I think is better done in person. …the patient interacting side of it has to be done through face to face training…"* (Participant 6, consenter)
Day or half-day training	*"Yeah, I mean there are all sorts of courses available. The vast majority of clinicians haven't got time to do it and so they're more likely to come to a half day or a one day training course than they are to sit down and do some on line training. [some clinicians] haven't got the time to take a year or two out to do a Masters, they want something much more quick and practical and off the shelf and ready now, that just gets them up to speed so that they know enough knowledge to know which test to offer to whom and when."* (Participant 14, genetic clinician)<br><br>*"I guess an open training session would be useful, in terms of something like an FAQ sort of session as to what are the expected questions from families and you know how to [answer] them, that sort of a training would be helpful."* (Participant 15, non-genetic clinician)
Training should be mandatory / national Standard / certification	*"Mandatory is a way of keeping on top of who's actually been trained, when they were trained and you get your refresher so, yeah, you do your basic, you get your certificate."* (Participant 10, consenter)
Genetic clinicians train non-genetic colleagues / Buddying systems and genomics champions	*"So what we're trying to set up is a kind of a buddying system where we will try and have clinical genetics working with sort of genomics champion in that speciality…. So that the genomics champion from the speciality who will put themselves forward as a sacrificial lamb, and the genetics consultant or genetics counsellor with experience, will kind of discuss and agree what the steps are with feeding that information back to patients. … So what's happened is our genetics teams have gone to the cancer MDTs, disease specific cancer MDTs and they're trying to help people understand about the difference between somatic mutations and germ line mutations. And whether they are clinically actionable… And I think that is working quite well and it's quite labour intensive for the genetics team, but they are you know, working with particular oncologists who want to learn all about this. It's that kind of partnership between genetics and then somebody who's prepared to be the genomics champion from the speciality."* (Participant 9, genetic clinician)

For peer review only

Study invitation sent to potential participants

Dear [name] ,

As you are aware we are running a research study looking at patient's views and experience of being offered whole genome sequencing through the 100,000 Genomes Project. As part of this study it is important to understand health professional experiences and views around offering whole genome sequencing. This research will help us to develop educational material for health professionals and for patients considering whole genome sequencing.

We would like to invite you to take part in an interview. If you would be willing to help with the study we will ask you to be interviewed either face-to-face at your place of work or by phone, whichever suits you. The interviews take around 30-45 minutes and will cover points such as your experience of discussing sequencing, consenting patients and delivering results and the potential barriers to informed consent and how can these be minimised.

The discussion will be audio-taped, the transcripts will be anonymised and your name will not be used in any reports or publications. Participation is, of course, voluntary and you can change your mind about being in the study at any time, without giving a reason. This research is organised by Research Teams at Great Ormond Street Hospital and UCL Institute of Child Health. The study is funded by the National Institute for Health Research.

If you are willing to take part in an interview or want to ask further questions, please let us know either by email or telephone.

If you would like any more information please let us know.

We look forward to hearing from you.

For peer review only

Interview guide for healthcare professionals

Background of participant and 100KGP training

1. What is your professional background? e.g. cardiologist, paediatrician etc
2. Had you offered whole genome sequencing in either a research or clinical capacity prior to the 100,000 Genomes Project beginning?
3. Can you briefly describe your role in the 100,000 Genomes Project?
4. Prior to recruiting participants into the 100,000 Genomes Project, did you have to undergo any type of training? If so what training did you undergo?

Recruitment into the 100KGP

5. Which of your patients do you discuss the 100KGP with?
6. Can you describe the discussion you have with those patients?
   a. What do you tell them about the 100KGP?
   b. What questions do they ask about the 100KGP?
   c. How long does that discussion tend to last?
   d. Have there been any difficulties you have encountered whilst discussing the 100KGP with patients?
7. If the patient is interested in taking part in the 100KGP what do you do next?
8. Have you had many people that decline to be referred into the 100KGP?
   a. What are their reasons for declining?
9. Have you come up against any problems recruiting into the study e.g. organisational issues or other difficulties?

Return of results in the 100KGP

10. Have you returned any results from the 100KGP to your patients?

    If participant HAS returned results

    a. Can you describe how you receive those results?
    b. What do you do once you have received those results? (write to patient? ask them to come to a clinic appointment?)
11. What kinds of results have you given to patients? (Primary findings; secondary findings)
12. What has the experience of returning results from WGS been like for you?
    a. Do you feel adequately prepared to discussion WGS results with patients?
    b. Have you encountered any difficulties?

For peer review only

c. Are you comfortable returning secondary findings?

13. What are patients' reactions to receiving results?

    a. What type of questions to patients ask?

    b. What concerns if any do they have?

    c. Do you feel able to address any concerns that they have?

If participant HAS NOT returned results

14. Do you know how the results will be sent to you?

    a. How will you contact the patient?

    b. Do you feel adequately prepared to discuss WGS results?

    c. Are you comfortable with returning secondary findings?

    d. Can you anticipate any difficulties?

Moving WGS into routine clinical practice

15. What do you think about whole genome sequencing being offered in routine clinical practice after the 100,000 Genomes Project has been completed?

    a. What do you see as the main advantages, benefits?

    b. What concerns, if any, do you have about offering whole genome sequencing being offered in routine clinical practice?

    c. In your view, what (if any) are the main limitations of using whole genome sequencing for routine clinical practice?

    d. In your view, what (if any) are the main challenges of offering whole genome sequencing in routine clinical practice?

16. Is there anything you would like to change about how whole genome sequencing is offered?

17. What advice would you give to other healthcare professionals discussing whole genome sequencing with patients?

18. What are your thoughts about how we would train health professionals that might be expected to offer whole genome sequencing in the future?

For peer review only

Interview guide for consenters

Background of participant and 100KGP training

19. What is your professional background? e.g. genetic counsellor, research fellow etc
20. What experience did you have of offering genetic testing or sequencing to patients prior to your involvement in the 100,000 Genomes Project?
    a. Specifically, had you offered whole genome sequencing prior to the 100,000 Genomes Project beginning?
21. Do you have any other relevant experience that you have brought to the role, e.g. consenting patients for research?
22. Can you briefly describe your role in the 100,000 Genomes Project?
23. Prior to recruiting participants into the 100,000 Genomes Project, what training did you undergo?
    a. Training specifically provided by the 100,000 Genomes Project
    b. Other training
24. What are your thoughts on the training that you did?
    a. Do you have any thoughts about the way it was delivered?
    b. Did you feel it adequately prepared you to recruit into the 100KGP?
    c. Did you feel anything was missing / could be improved?

Recruitment into the 100KGP

25. What has been your experience of recruiting patients into the 100KGP?
    a. What aspects of recruitment have worked well?
    b. What aspects of recruitment haven't worked well?
    c. How might recruitment be improved in your opinion?
26. Have you come up against any other problems recruiting into the study e.g. organisational issues or other difficulties?

Assessing informed consent in the 100KGP

27. What is your approach to consenting patients into the 100KGP?
    a. How do you generally begin the conversation?
    b. Are there set ways you conduct the session i.e. when you go through the consent form, family history etc or does it alter depending on the participants?

For peer review only

   c. Does the discussion tend to be led by you or by the patient?

28. When you are talking to patients about consenting to the 100,000 Genomes Project, do you try to explore their understanding of the Project?

   a. If yes, how do you do this? Do you use any particular techniques to elicit patients' knowledge and understanding? What are they?

29. When you are talking to patients about consenting to the 100,000 Genomes Project, do you try to explore their attitudes, views, preferences, concerns around the Project?

   a. If yes, how do you do this? Do you use any particular techniques to elicit patients' attitudes and/or concerns? What are they?

30. What (if any) do you think are the main barriers to helping patients make informed choices about taking part in the 100,000 Genomes Project? (length of appointment; cultural barriers; lack of tools to support the consent discussion)

31. How does the method of contact i.e. whether they know about the 100KGP when they arrive at the hospital or whether they are recruited unexpectedly following another appointment, impact the consent discussion?

   a. Does it make a difference if they have already received written information about the study prior to the consent appointment?

Participants experiences in the 100KGP

32. What are the common questions patients have about taking part in the study?

   a. What concerns do patients have about taking part?

   b. What are their reasons for declining to take part?

33. Which are the main aspects of the study that you feel you spend most time discussing with patients (e.g. what genome sequencing is, secondary findings, accessing data etc)?

34. Do you think patients are putting a lot of thought into the decision to accept/decline testing? Do any want additional time to think about it?

35. What has been your experience discussing secondary findings with patients?

   a. Is it something that generates discussion and if so what kind of discussion?

   b. Who is the discussion between? (patient / parents themselves? Patient and health professional?)

36. Have you had experience of receiving consent or assent from a patient under the age of 16? What has been your experience?

37. Have you had to reconsent anyone turning 16 to remain in the project (with capacity)? What has been your experience of doing this?

   a. Has anyone withdrawn?

For peer review only

Moving WGS into routine clinical practice

38. What do you think about whole genome sequencing being offered in routine clinical practice after the 100,000 Genomes Project has been completed?

    a. What do you see as the main advantages, benefits?

    b. What concerns, if any, do you have about offering whole genome sequencing being offered in routine clinical practice?

    c. In your view, what (if any) are the main limitations of using whole genome sequencing for routine clinical practice?

    d. In your view, what (if any) are the main challenges of offering whole genome sequencing in routine clinical practice?

39. Is there anything you would like to change about how whole genome sequencing is offered?

40. What advice would you give to other healthcare professionals discussing whole genome sequencing with patients?

41. What are your thoughts about how we would train health professionals that might be expected to offer whole genome sequencing in the future?

For peer review only

Standards for Reporting Qualitative Research (SRQR)*

http://www.equator-network.org/reporting-guidelines/srqr/

Title and abstract

	Page/line no(s).
**Title** - Concise description of the nature and topic of the study Identifying the study as qualitative or indicating the approach (e.g., ethnography, grounded theory) or data collection methods (e.g., interview, focus group) is recommended	1
**Abstract** - Summary of key elements of the study using the abstract format of the intended publication; typically includes background, purpose, methods, results, and conclusions	2

Introduction

**Problem formulation** - Description and significance of the problem/phenomenon studied; review of relevant theory and empirical work; problem statement	3
**Purpose or research question** - Purpose of the study and specific objectives or questions	3

Methods

**Qualitative approach and research paradigm** - Qualitative approach (e.g., ethnography, grounded theory, case study, phenomenology, narrative research) and guiding theory if appropriate; identifying the research paradigm (e.g., postpositivist, constructivist/ interpretivist) is also recommended; rationale**	6
**Researcher characteristics and reflexivity** - Researchers' characteristics that may influence the research, including personal attributes, qualifications/experience, relationship with participants, assumptions, and/or presuppositions; potential or actual interaction between researchers' characteristics and the research questions, approach, methods, results, and/or transferability	6
**Context** - Setting/site and salient contextual factors; rationale**	5
**Sampling strategy** - How and why research participants, documents, or events were selected; criteria for deciding when no further sampling was necessary (e.g., sampling saturation); rationale**	5
**Ethical issues pertaining to human subjects** - Documentation of approval by an appropriate ethics review board and participant consent, or explanation for lack thereof; other confidentiality and data security issues	5
**Data collection methods** - Types of data collected; details of data collection procedures including (as appropriate) start and stop dates of data collection and analysis, iterative process, triangulation of sources/methods, and modification of procedures in response to evolving study findings; rationale**	5-6

For peer review only

For peer review only - http://bmjopen.bmj.com/site/about/guidelines.xhtml

**Data collection instruments and technologies** - Description of instruments (e.g., interview guides, questionnaires) and devices (e.g., audio recorders) used for data collection; if/how the instrument(s) changed over the course of the study	5-6
**Units of study** - Number and relevant characteristics of participants, documents, or events included in the study; level of participation (could be reported in results)	7
**Data processing** - Methods for processing data prior to and during analysis, including transcription, data entry, data management and security, verification of data integrity, data coding, and anonymization/de-identification of excerpts	6
**Data analysis** - Process by which inferences, themes, etc., were identified and developed, including the researchers involved in data analysis; usually references a specific paradigm or approach; rationale**	6
**Techniques to enhance trustworthiness** - Techniques to enhance trustworthiness and credibility of data analysis (e.g., member checking, audit trail, triangulation); rationale**	6

Results/findings

**Synthesis and interpretation** - Main findings (e.g., interpretations, inferences, and themes); might include development of a theory or model, or integration with prior research or theory	7-11
**Links to empirical data** - Evidence (e.g., quotes, field notes, text excerpts, photographs) to substantiate analytic findings	7-11 & Tables

Discussion

**Integration with prior work, implications, transferability, and contribution(s) to the field -** Short summary of main findings; explanation of how findings and conclusions connect to, support, elaborate on, or challenge conclusions of earlier scholarship; discussion of scope of application/generalizability; identification of unique contribution(s) to scholarship in a discipline or field	12
**Limitations** - Trustworthiness and limitations of findings	14

Other

**Conflicts of interest** - Potential sources of influence or perceived influence on study conduct and conclusions; how these were managed	15
**Funding** - Sources of funding and other support; role of funders in data collection, interpretation, and reporting	17

*The authors created the SRQR by searching the literature to identify guidelines, reporting standards, and critical appraisal criteria for qualitative research; reviewing the reference lists of retrieved sources; and contacting experts to gain feedback. The SRQR aims to improve the transparency of all aspects of qualitative research by providing clear standards for reporting qualitative research.

For peer review only - http://bmjopen.bmj.com/site/about/guidelines.xhtml

**The rationale should briefly discuss the justification for choosing that theory, approach, method, or technique rather than other options available, the assumptions and limitations implicit in those choices, and how those choices influence study conclusions and transferability. As appropriate, the rationale for several items might be discussed together.

Reference:

O'Brien BC, Harris IB, Beckman TJ, Reed DA, Cook DA. **Standards for reporting qualitative research: a synthesis of recommendations.** *Academic Medicine*, Vol. 89, No. 9 / Sept 2014 DOI: 10.1097/ACM.0000000000000388

For peer review only

For peer review only - http://bmjopen.bmj.com/site/about/guidelines.xhtml
